# Bacterial nanotechnology as a paradigm in targeted cancer therapeutic delivery and immunotherapy

**DOI:** 10.1038/s41378-024-00743-z

**Published:** 2024-08-20

**Authors:** Ahmad Gholami, Milad Mohkam, Saeede Soleimanian, Mohammad Sadraeian, Antonio Lauto

**Affiliations:** 1 Biotechnology Research Center, Shiraz University of Medical Sciences, Shiraz, Iran; 2 Department of Pharmaceutical Biotechnology, Shiraz University of Medical Sciences, Shiraz, Iran; 3 Allergy Research Center, Shiraz University of Medical Sciences, Shiraz, Iran; 4 Institute for Biomedical Materials and Devices (IBMD), Faculty of Science, University of Technology Sydney, Sydney, NSW 2007 Australia; 5 School of Science, University of Western Sydney, Campbelltown, NSW 2560 Australia; 6 School of Medicine, University of Western Sydney, Campbelltown, NSW 2560 Australia

**Keywords:** Nanoscience and technology, Nanobiotechnology

## Abstract

Cancer, a multifaceted and diverse ailment, presents formidable obstacles to traditional treatment modalities. Nanotechnology presents novel prospects for surmounting these challenges through its capacity to facilitate meticulous and regulated administration of therapeutic agents to malignant cells while concurrently modulating the immune system to combat neoplasms. Bacteria and their derivatives have emerged as highly versatile and multifunctional platforms for cancer nanotherapy within the realm of nanomaterials. This comprehensive review delves into the multifaceted and groundbreaking implementations of bacterial nanotechnology within cancer therapy. This review encompasses four primary facets: the utilization of bacteria as living conveyors of medicinal substances, the employment of bacterial components as agents that stimulate the immune system, the deployment of bacterial vectors as tools for delivering genetic material, and the development of bacteria-derived nano-drugs as intelligent nano-medications. Furthermore, we elucidate the merits and modalities of operation pertaining to these bacterial nano-systems, along with their capacity to synergize with other cutting-edge nanotechnologies, such as CRISPR-Cas systems. Additionally, we offer insightful viewpoints regarding the forthcoming trajectories and prospects within this expanding domain. It is our deduction that bacterial nanotechnology embodies a propitious and innovative paradigm in the realm of cancer therapy, which has the potential to provide numerous advantages and synergistic effects in enhancing the outcomes and quality of life for individuals afflicted with cancer.

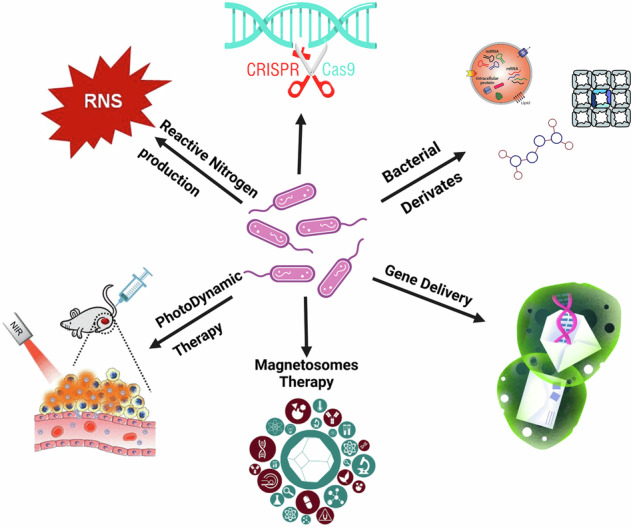

## Introduction

Bacterial therapy and nanotherapy have emerged as promising approaches in the field of cancer treatment, offering distinct advantages and limitations. Whether used alone or in combination with conventional methods, bacterial therapy has demonstrated effectiveness in regressing tumors and inhibiting metastasis^1^. This therapeutic approach can directly target cancer cells through oncolytic and cytotoxic activities or modulate the immune system to impede tumor growth^2^. By engineering bacteria, it is possible to enhance their ability to deliver immunomodulators, thereby boosting antitumor immunity while ensuring safety. Notable bacteria used as immunotherapeutic agents include *Bifidobacterium*^3^, *Clostridium novyi*^4^, *Listeria monocytogenes*^5^, and *Salmonella typhimurium*^6^. Moreover, various bacterial components such as proteins, spores, toxins, and peptides can indirectly serve as adjuvants to activate the immune system^7^.

Concurrently, nanomedicines have been developed as an alternative method for treating cancer, enabling precise targeting of tumor tissues^8^. Nanotherapy facilitates the delivery of drugs directly to the desired site, thereby increasing drug concentration while minimizing adverse effects on healthy cells^9^. However, nanotherapy encounters significant challenges arising from the body’s defense mechanisms and tumor microenvironment anomalies, which hinder the effective delivery of nanotherapeutics to tumors^10–12^.

**Fig. 1:**
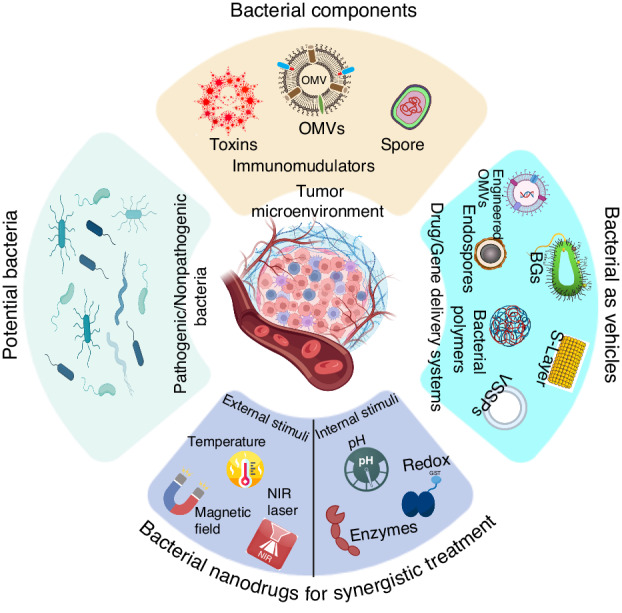
A summary of how bacteria and their nano-drugs can be used to fight cancer. Created with BioRender.com

Recent advancements in nanotechnology research have paved the way for integrating bacterial therapy into cancer treatment, offering innovative strategies. Bacteria and their derived nano-drugs exhibit tremendous potential for addressing the challenges associated with cancer treatment. These advancements have led to the development of bacterial nanovesicles, drug delivery systems based on nanotechnology, bacterial membrane-coated nanoparticles, and hybrid bacteria-nanoparticle systems (Fig. 1)^13^.

This review explores the potential of bacterial nanotechnology in targeted cancer therapeutic delivery and immunotherapy, focusing on four key aspects: bacteria, bacterial constituents, bacterial vectors, and bacteria-derived nano-drugs. We highlight how bacteria-nanoparticle hybrid systems can deliver drugs and genes to cancer cells, how they work, their advantages, and their nanoparticle functions. In addition, we focus on new gene delivery systems in bacterial nanotechnologies, such as CRISPER-CAS systems. However, we also address the challenges faced by bacterial nanotechnology in cancer therapy and discuss future perspectives in this innovative field. Overall, the review emphasizes the importance of bacterial nanotechnology as a promising approach to cancer treatment.

## Types of hybrid bacterial nano-systems

The hybrid bacterial nano-systems integrate living bacteria with non-living structures such as nanoparticles to expand the antitumor applications of bacteria. By working together, the biohybrid system achieves higher levels of functionality than each component can achieve individually. However, systemic injections, such as intravenous injections, can cause serious systemic inflammation and be cleared by antibodies, posing challenges to living organisms. To address this issue, nanomaterial surface modification of bacteria offers a protective approach to reducing systemic inflammation^14^. Furthermore, these bio-hybrids are able to transport a variety of biomedical cargo (photothermal drugs, chemotherapeutics, immunotherapeutics, or photodynamic drugs) to tumor tissue. Once the cargo is delivered, it can be released locally within the tumor to ensure optimal efficacy and minimal adverse effects. Biohybrids can also release drugs under specific tumor conditions such as marginally acidic pH, specific enzymes, and ROS^15^.

**Fig. 2:**
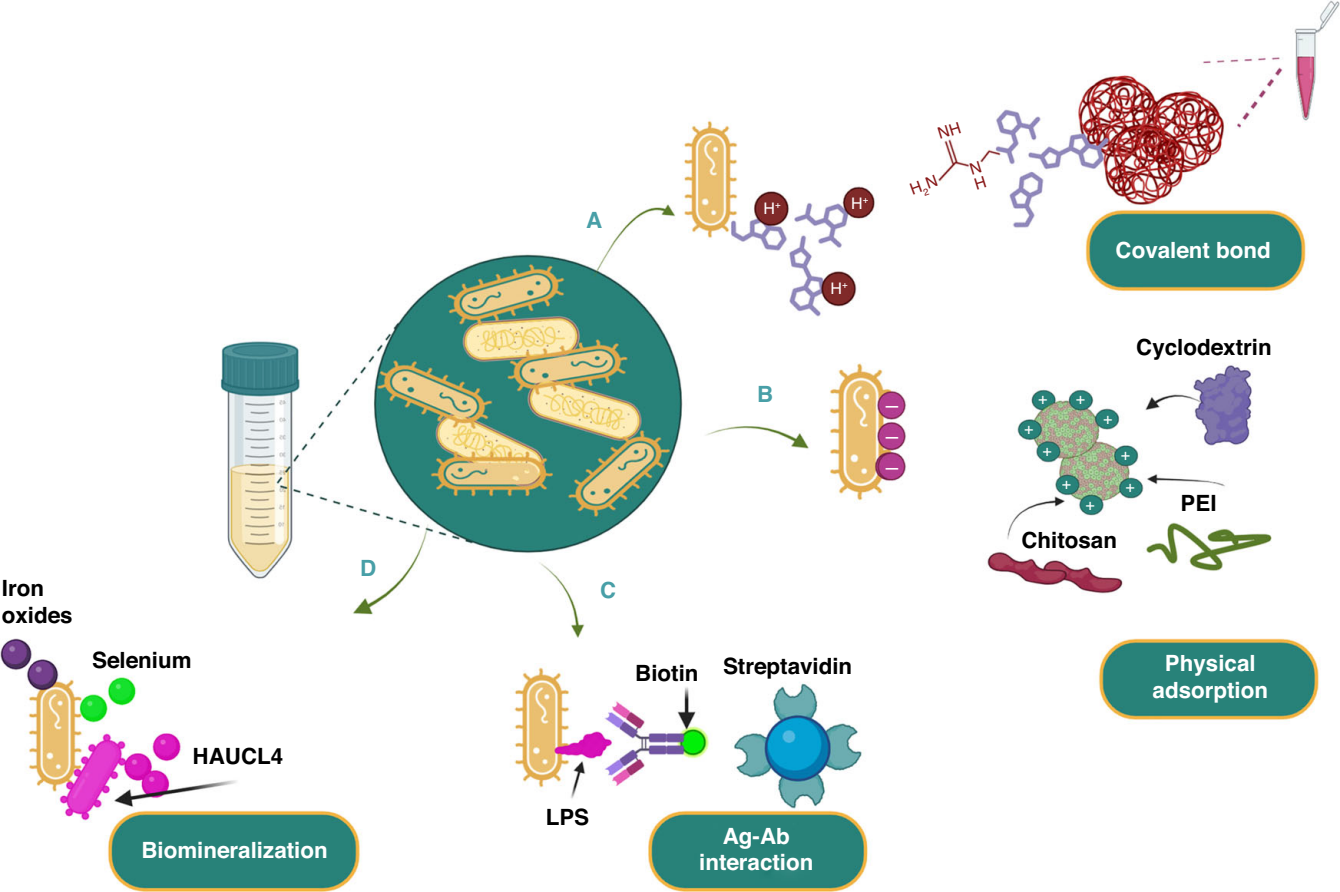
The diagram of the process of creating bacteria-nanoparticle bio-hybrids using various methods of binding, such as physical adsorption, biomineralization, chemical bonds, and other methods. Covalent bonds are formed by connecting groups on the bacteria and nanoparticle surfaces. Physical adsorption happens when negatively charged bacteria and positively charged nanostructure (often covered with cationic polymers like PEI and chitosan), stick together. Biomineralization is the process of trapping and changing metal ions into metal elements on the bacterial surface^211^. Other methods of attaching are also possible. Biomineralization involves capturing and converting metal ions into metal elements on the surface of bacteria. Other forms of binding are also possible—abbreviations: LPS, lipopolysaccharides; PEI, polyethyleneimine; MOF, metal-organic framework. Created with BioRender.com

The following section will examine the mechanisms of bacterial adhesion and drug encapsulation, their efficacy, and their applications in oncology. These features are illustrated in Fig. 2.

### Covalent Bond

Bacteria are living organisms that are ideal for chemical modification because they have different chemical groups on their surface, such as amino groups (-NH_2_) that are part of the proteins in the bacterial cell membrane. Certain nanoparticles can be chemically conjugated with bacteria by attaching reactive groups (-COOH, -CHO) to form biohybrids through the formation of either amide or imine bonds on their surface, including amino groups (-NH2) that are part of the proteins in the bacterial cell membrane. These functional groups serve as attachment sites for chemical modification^14,16^. For example, nanoparticles loaded with indocyanine green (ICG) were attached to the surface of *S. typhimurium* YB1 strain by amide bonds to make YB1-INPs. YB1-INPs showed great ability to target tumors and photothermal effects, leading to a 14-fold increase in accumulation in the tumor and considerable tumor removal with no regression^17^. The observed effect may be attributed to the motility of YB1, enabling it to penetrate deeper into the tumor tissue and facilitate the distribution of ICG-loaded nanoparticles throughout the tumor mass^17^. The incorporation of carboxyl groups in the nanoparticles was the most commonly used approach to link nanoparticles to bacteria and provided enhanced stability in vivo^17^. Moreover, the use of imine bonds to form biohybrids can facilitate the selective detachment of nanoparticles from bacteria in acidic tumor microenvironments without affecting nanoparticle absorption by cells^17^. In another study, Luo et al. adhered nanoparticles containing perfluorohexane (PFC) to the surface of *B.longum*^18^. In this context, this bacterium likely exhibits chemotaxis towards the tumor microenvironment driven by specific chemical gradients. This directed movement allows the bacteria to deliver the PFC-loaded nanoparticles directly to the tumor site, improving the curative efficacy of high-intensity focused ultrasound (HIFU) therapy. HIFU is a non-invasive therapeutic modality that utilizes focused ultrasound waves to generate heat within targeted tissues. The presence of PFCs within the tumor enhances the effectiveness of HIFU therapy by converting the sound energy into heat more efficiently, leading to localized thermal ablation of cancer cells^18^. However, the use of imine bonds to form biohybrids was found to be less stable in acidic tumor microenvironments, resulting in the separation of nanoparticles from bacteria due to hydrolysis^19^. Nevertheless, this approach could facilitate the selective detachment of nanoparticles from bacteria in tumor microenvironments without affecting nanoparticle absorption by cells. This potentially facilitates the controlled release of PFC nanoparticles within the tumor, maximizing their availability for HIFU therapy. Furthermore, the detached nanoparticles might still be retained within the tumor due to the Enhanced Permeability and Retention (EPR) effect, leading to further enhancing therapeutic efficacy^19^. In a similar study, Chen et al. created nanoparticles loaded with photosensitizer, utilizing Zeolite imidazole framework (ZIF-90)^20^. They subsequently synthesized an imine bond among the bacterium’s amino group and the nanoparticles’ aldehyde group to alter *Shewanella* mR-1. Similar to the previous research, *Shewanella* mR-1 utilizes its inherent ability for chemotaxis to navigate towards the tumor site due to the characteristic chemical gradients present in the tumor microenvironment. In this context, the photosensitizer-loaded ZIF-90 was able to separate from the bacterial surface and exert photodynamic (PDT) and photothermal (PTT) anticancer activity upon reaching acidic tumor tissues and being irradiated by a laser^20,21^. In this context, these activated photosensitizers can generate cytotoxic reactive oxygen species (ROS) within tumor cells, inducing cell death. Additionally, they can convert light energy into heat, thereby facilitating photothermal therapy (PTT) in conjunction with photodynamic therapy (PDT), thereby augmenting therapeutic effectiveness^20,21^.

Magnetotactic bacteria are a distinct bacterial phylum capable of biosynthesizing magnetosomes consisting of iron oxide magnetic nanoparticles. The magnetosomes of magnetotactic bacteria are arrayed along their length like compasses and are used to guide the bacteria to their targets^22–24^. *Magnetococcus marinus* strain MC-1 is an example of a magneto-aerotactic bacterium that has been utilized to deliver liposomes containing SN-38 drug into the hypoxic zone of tumors^25^. SN-38 is the primary active metabolite of the chemotherapeutic agent CPT-11 which exerts its effects by inhibiting the enzyme DNA topoisomerase I, a pivotal player in DNA replication and repair, particularly in rapidly dividing cells such as cancer cells. Through this inhibition, SN-38 disrupts the normal course of DNA replication, inducing DNA damage and ultimately triggering cell death via apoptosis, while also eliciting cell cycle arrest. Moreover, SN-38-induced apoptosis in cancer cells can prompt immunogenic cell death, characterized by the release of specific signals and molecules that activate the immune system. Consequently, immune cells such as cytotoxic T lymphocytes (CTLs) are recruited to recognize and eliminate residual cancer cells, enhancing the therapeutic efficacy against malignancies^25^.

Covalent bonding among the DSPE-PEG-COOH carboxyl and the amino groups on the surface of the bacterium is required for liposome attachment to bacteria. This allowed the bacteria to be directed toward hypoxic regions of tumors using magnetotactic control^25,26^. Moreover, by applying an external magnetic field, the researchers can direct the bacteria carrying liposomal drugs towards the tumor site^25^. In this regard, the mean tumor targeting ratio was greater than 50%, indicating that targeted chemotherapy is becoming more effective. This delivery technique can potentially improve the efficacy of many other therapy modalities, such as the delivery of photodynamic sensitizers or radio-sensitizers to hypoxic regions of tumors^25^. Another study evaluated the effect of attaching liposomes to the surface of magnetotactic bacteria on their swimming speed. The findings of the study indicate a reduction of 27% in the swimming velocity of the biohybrid, accompanied by an increase in velocity under identical magnetic field conditions^27^. This confirms the efficacy of magnetotactic bacteria in facilitating the chemical bonding of nanoparticles, thereby enabling them to overcome diffusion barriers within solid tumors^28^.

Besides direct attachment, bacteria-nanomaterial hybrids can be formed through direct attachment or chemical modification. Kuru et al. developed a universal approach to changing the bacterial surface by incorporating diverse D-amino acids of varying sizes and features into peptidoglycan (PG) attached to the bacteria^29^. The D-amino acid backbone contains 4-chloro-7-nitrobenzofurazan (NBD-Cl) and 7-hydroxycoumarin 3-carboxylic acid (HCC-OH), enabling the spatiotemporal tracing of the biosynthesis of PG within the cell wall^29^. This approach creates additional chemical sites for nanomaterial attachment. Typically, this process involves modifying the bacteria with azide groups, followed by alkyne-strained modifications on the nanomaterials. In this way, the two functional groups can establish triazole bonds through the click reaction^19^. In parallel, Moreno et al. have also demonstrated that nanoparticles (mesoporous silica (MSN)) loaded with drugs can be attached to *E. coli*’s surface. This approach results in improved penetration into the tumor matrix and homogeneous drug distribution across tumor tissues; however, some indirect immunomodulation can occur through immunogenic cell death and potential alterations to the tumor microenvironment. Further research is needed to fully understand the interplay between this bacterial nanotechnology approach and the immune system^30^.

Compared to other bioconjugation methods, covalent bonds are the strongest type of chemical bond since their dissociation enthalpy is greater than 300 kJ/mol^31^. According to the in-vivo investigations, it is indicated that the biohybrid entity will exhibit stability subsequent to its reaching the tumor site^32^.It is necessary for future research to explore whether nanomaterials detach from bacteria within the cellular milieu or if nanomaterial/bacterial conjugates are capable of being internalized via non-phagocytic cells.

### Physical adsorption

In addition to covalent modification, physical adsorption has been utilized to create nanoparticle-bacteria biohybrids through electrostatic, van der Waals, hydrophobic forces, and hydrogen bonds. The reversal of the negative to the positive potential of nanomaterial compounds has been achieved through cationic polymers or protonation. This method effectively overcomes the negative potential of bacteria surfaces, enabling the formation of hybrids through electrostatic adsorption forces. For example, polyethylenimine (PEI), a cationic polymer, has been used to modify and absorb nanoparticles onto the bacterial surface^19^. PEI is characterized by a high density of amino groups (-NH2), which readily form bonds with various functional groups present on bacterial surfaces. Serving as a molecular bridge, PEI facilitates interactions between the negatively charged bacterial surface and nanoparticles. Leveraging its positive charge, PEI establishes electrostatic attractions with negatively charged nanoparticles, thereby aiding their adhesion to bacterial surfaces^19^. This phenomenon enables bacteria to effectively transport nanoparticles loaded with therapeutic agents to tumor sites. Additionally, PEI may independently contribute to the uptake of nanoparticles by cancer cells. Its positive charge enables interactions with the negatively charged cell membrane, potentially facilitating the internalization of nanoparticle-PEI complexes into cells. From an immunological standpoint, PEI is not inherently engineered to directly stimulate the immune system. However, in certain scenarios, the immune system may identify and endeavor to eliminate PEI-modified bacteria^19^.

**Fig. 3:**
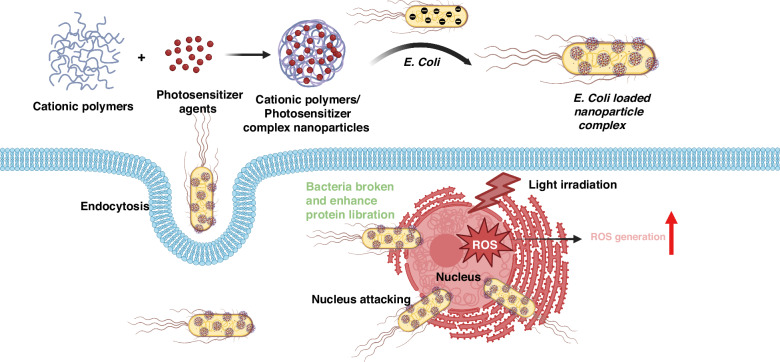
The method for using of live *E. coli* coated with photosensitizer nanoparticles on bacteria followed by intracellular transportation of live *E. coli* coated with nanoparticle-coated and photosensitizer delivery. Created with BioRender.com

In one study, Wu et al. incorporated a photosensitizer into a lipid nanoparticle and coated it onto *E. coli* using PEI (600 Da). This resulted in a multifunctional hybrid with an increased ability to invade cancer cells and effectively induce light-mediated tumor cell death (Fig. 3)^33^. In a similar work, Hu and Chen, developed a DNA vaccine. This vaccine was designed to express the vascular endothelial growth factor receptor (VEGFR2) gene. They achieved this by using electrostatic self-assembly techniques with pDNA and β-cyclodextrin-PEI to form nanoparticles. These nanoparticles were then affixed to the surface of invading *Salmonella* through electrostatic interactions^34,35^. This DNA nano-vaccine stimulated the activation of cytotoxic T lymphocytes for immunotherapy, impeding the initiation of cancerous blood vessels by disrupting the VEGFR2 pathway, a receptor protein crucial in the angiogenic process of tumor neovascularization. This intervention led to comprehensive tumor suppression by disrupting the formation of new blood vessels that provide essential nutrients and oxygen to the tumor, thereby inducing a state of nutrient deprivation and impeding tumor progression^34,35^. The immune-stimulating activity of bacteria can serve as adjuvants in the tumor microenvironment, intensifying the activating effect of the immune system by activating antigen-presenting cells (APCs) like dendritic cells leading to boosting the overall effectiveness of the DNA vaccine. In addition to chitosan and cationic peptides, other cationic polymers can also bind complexes via positively charged nanoparticles. Luo et al. fabricated nanorods with an imaging agent and protonated oleic acid, which was then electrostatically coupled with *B. breve* UCC2003^36^. Due to bacteria targeting, a high concentration of imaging agents was located at tumor sites to enhance fluorescence signal amplitude.

Molecules can cluster into steady structures on membrane surfaces through a physical phenomenon known as supramolecular self-assembly. Researchers used this technique to deliver bacteria in the stomach and after four hours, approximately 90% of the bacteria detached effectively to reach the intestinal tract^37^. The supramolecular self-assembly approach can thus deliver the drug directly to the tumor site by embedding an anti-tumor agent into the bacteria. This approach has shown promising results for targeting cancer.

Physically adsorbed hybrids may be a more appropriate option for therapeutic agents; as passive adsorption can readily facilitate the formation of stable non-covalent interactions, including van der Waals and electrostatic forces. In contrast, covalent conjugations may not be stable in blood plasma for example. Furthermore, bacterial proliferation can lead to shedding nanoparticles from the surface, and losing the outer layer of bacteria in the blood stream will likely trigger an inflammatory response.

### Biomineralization

Biomineralization is the incorporation of mineral compounds into the matrix of living organisms. Certain bacteria can biomineralize nanoparticles via a biological enzymatic process involving the conversion of metal ions into metal elements. Researchers have recently developed a range of inorganic-bacteria matrices using biomimicry mineralization. Biomineralization holds significant promise for therapeutic delivery in cancer patient. This is because biomineralizing nanoparticles on bacteria’s surfaces would not interfere with the bacteria’s ability to target tumors. Biomineralization of bacteria has been carried out using various materials, such as silica, zinc-silicates, metal-organic frameworks (MOF), iron oxides, selenium, gold nanoparticles, and calcium phosphates.

**Fig. 4:**
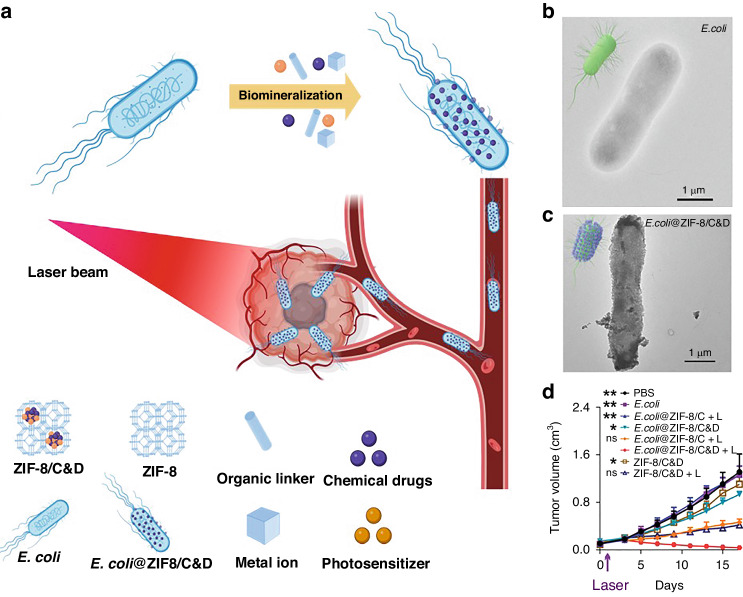
Mineralization of Tumor-Targeting *E. coli* for Enhanced Therapeutic Delivery Using ZIF-8. **a** Utilizing zeolitic imidazolate framework-8 (ZIF-8), tumor-targeting *E. coli* undergo biomimetic mineralization for effective therapeutic delivery. **b**, **c** Transmission electron microscopy (TEM) captures the primary *E. coli* and *E. coli*@ZIF-8/C&D structures. **d** Assessment of tumor growth in 4T1 tumor-bearing mice treated with various interventions, demonstrating distinct treatment outcomes (*p < 0.05, **p < 0.01). Adapted with permission from original source^42^. © 2020 WILEY-VCH Verlag GmbH & Co. KGaA, Weinheim

Several metals and metallic oxides have been shown to be effective photothermal agents for photothermal therapy (PTT), such as the tetrapyrrolic derivatives of palladium (II) (WST11), Sn (IV) (Purlytin), and Lu (III) (Lutex)^38^. The targeting capability of PTT has been enhanced by using specific bacteria to synthesize these photothermal agents. Chen et al. reported that *Shewanella oneidensis* MR-1, can reduce sodium tetra-chloropalladate (Na_2_PdCl_4_) into Pd nanoparticles^20,39^. As a self-mineralizing photothermal bacterium, it exhibits a natural propensity to selectively target tumors; migrating towards them and penetrating deeper into their tissue compared to externally administered nanoparticles. This enhanced penetration facilitates more efficient photothermal activity within the tumor microenvironment^40^. Wang et al. used *Shewanella algae* K3259 to synthesize gold nanoparticles on the bacterium’s surface, resulting in improved bacterial metabolism as well as photodynamic therapy targeting gold nanoparticles. By transferring photoelectrons generated by AuNPs into the bacterial cytoplasm, AuNPs were able to increase the production of antitumor tetrodotoxin^41^. The mechanism by which gold nanoparticles (AuNPs) augment antitumor tetrodotoxin production in Shewanella algae K3259 is currently under investigation, with several potential explanations. AuNPs may serve as electron conduits, thereby enhancing energy production for tetrodotoxin biosynthesis. Alternatively, they could modulate gene expression pathways involved in toxin production. Localized alterations in the bacterial microenvironment near the AuNPs or direct interaction with biosynthetic enzymes are also plausible factors contributing to this phenomenon^41^. Yan et al. created a hybrid of *E. coli* and zeolitic imidazolate framework-8 layer (MOF) through biomineralization to broaden the range of loading types^42^. The study involved the loading of chemotherapeutic drugs (doxorubicin, D) and photosensitizers (chlorin e6, C) into a Metal-Organic Framework (MOF). The results showed that the MOF exhibited a superior therapeutic function due to the synergistic effect of the loaded substances. The method allows for incorporating various pharmaceuticals into drug delivery vehicles synthesized on bacterial surfaces. Examples of such vehicles include silica nanoparticles, MOF, calcium phosphate, and calcium carbonate nanoparticles (Fig. 4)^42^.

Recent advances in biomineralization have shown that coupling inorganic nanoparticles with bacteria can provide drug delivery systems with unique features, including the ability to convert magnetic forces and photothermal energy. The universality of this approach is restricted to the specific amino acids on the living organism’ surfaces. For this reason, further investigations are required to overcome these limitations^43^.

### Other binding forms

Various attachment methods have been employed in research, such as the use of bio-affinity bacteria-nanoparticle hybrids. These hybrids utilize moderately close binding forces, inherent interaction forces in biological systems^44^.

**Fig. 5:**
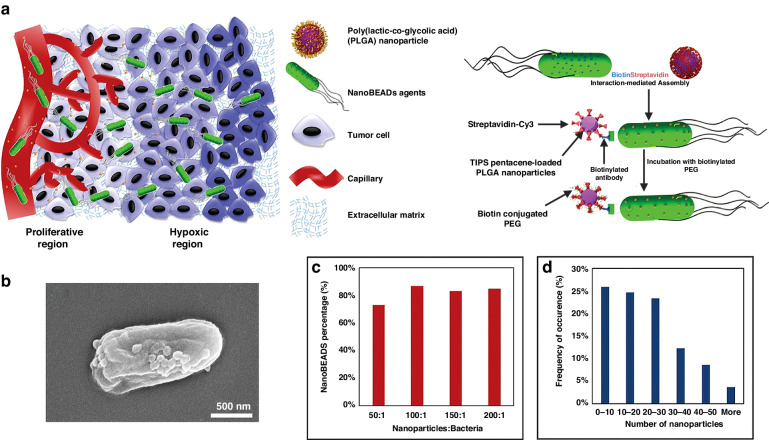
Development of a novel autonomous drug delivery system, NanoBEADS, through biotin-streptavidin conjugation. **a** Visualization of Enhanced NanoBEADS Infiltration into Poorly Vascularized Tumor Tissue in Comparison to Passive Nanoparticles (Left). Each NanoBEADS construct is created by linking multiple streptavidin-coated PLGA nanoparticles with a tumor-targeting biotinylated-antibody-coated *S. Typhimurium* VNP20009 using noncovalent affinity-based bonds between streptavidin and biotin. Subsequently, NanoBEADS assembly involved incubation with mPEG-biotin to neutralize any remaining streptavidin binding sites on the nanoparticles. **b** A Representative Scanning Electron Microscopy (SEM) Image of a NanoBEADS Construct. **c** Assessment of the Incidence of NanoBEADS Formation at Different Ratios of Nanoparticles to Bacteria Employed in NanoBEADS Fabrication. **d** Analysis of the Distribution of Nanoparticle Loading in NanoBEADS Constructs Produced at a Nanoparticle-to-Bacteria Ratio of 100:1 (*n* = 80). Adapted from original source^46^. Published by WILEY‐VCH Verlag GmbH & Co. KGaA, Weinheim. This is an open access article distributed under the terms of the Creative Commons CC BY license

Streptavidin and biotin represent one of the strongest protein-ligand interactions in biological systems. Streptavidin is a tetrameric biotin-binding protein known for its high degree of specificity in capturing biotin, making it an excellent tool for targeting drug delivery systems^45^. In one such approach, the bacterial outer membrane was targeted by biotin-labeled antibodies through incubation. Subsequently, the nanoparticle surfaces were covalently bonded to streptavidin. The resulting hybrid was generated by the process of co-incubation between biotin-labeled bacteria and streptavidin-coated nanoparticles. Sahari et al. utilized a biotin-labeled goat polyclonal antibody and *E. coli* MG1655m bacteria that specifically binds to lipopolysaccharides (LPS) e.g. lipid A. This allowed them to affix streptavidin-coated polymeric microparticles onto the surface of the bacteria (53). Poly(lactic-co-glycolic acid) nanoparticles were generated and affixed to the exterior of *S. typhimurium* VNP20009 using the identical method. The study found that the intratumoral transport of bacteria was not impeded by nanoparticle conjugation. Additionally, a noteworthy 100-fold enhancement in nanoparticle’s distribution and retention in solid malignancy was detected^46^ (Fig. 5). In a similar study, Uthaman et al. detailed the creation of a *S. typhimurium* strain that has been genetically modified to express biotin. This modification was made with the intention of enabling the bacteria to interact with streptavidin-conjugated microbeads in an anaerobic environment. The streptavidin on the HA beads and the biotin on the bacteria interacted with each other, resulting in an improved targeted anticancer treatment. This interaction enhanced chemotactic and biological targeting^47^. The surface attachment of bacteria to the microbeads was manipulated to regulate the collective self-propulsion force of bacteria, thereby facilitating the forward movement of the beads.

An interesting alternative to the streptavidin-biotin affinity system for delivering functional nanoparticles is the use of antibody-antigen interactions. Monoclonal antibodies can be used to coat nanoparticles, which can then be targeted to specific bacteria. One approach to achieve this is the use of an antibody-guided approach, as demonstrated by Luo et al.^36^. The research conducted in this study focused on administering *Clostridium difficile* spores directly into tumors in conjunction with the administration of nanoparticle-antibody engineered to target spore germination selectively. This approach takes advantage of the precise and targeted interactions facilitated by antibodies and antigens. Additionally, it leverages the bacterium as a navigational tool to guide nano-medicines toward tumors and facilitate the formation of hybrids within in-vivo conditions.

## Bacterial nanotechnology in cancer therapy

Bacterial nanotechnology refers to the utilization of bacteria or their constituent elements in the fabrication of nanoscale structures and devices, serving diverse purposes. Bacteria are being explored for example as potential natural or engineered vehicles for delivering therapeutic agents to tumor sites. In this section, we review recent developments and drawbacks in the field of bacterial nanotechnology for cancer treatment as well as compare them to current standard cancer therapies. The discussion primarily revolves around several key areas, namely bacterial toxins, nano-platforms derived from bacterial outer membranes, hybrid bacterial nano-systems used as vehicles for delivering chemotherapy drugs, very small-size proteoliposomes (VSSPs), the utilization of bacterial S-layer as a carrier for therapeutic agents, bacterial biopolymers, and bacterial ghosts. We also explore the potential advantages and constraints associated with bacteria agents in nanomedicine highlighting future prospects and developments in this rapidly evolving field.

### Bacterial nanotechnology vs. standard cancer therapies

Bacterial nanotechnology in cancer therapy represents a cutting-edge approach that offers unique advantages and challenges compared to current standard cancer therapies. To provide a comprehensive comparative analysis, we will delve into the relative benefits, limitations, and the specific niche that bacterial nanotechnology could potentially occupy in the landscape of cancer treatment.

#### Current standard cancer therapies

Traditional cancer treatments encompass a range of modalities such as chemotherapy, radiation therapy, targeted therapy, and immunotherapy. While these approaches have been instrumental in cancer management, they come with inherent limitations. Chemotherapy, for instance, lacks specificity and often leads to systemic toxicity due to its non-selective nature. Radiation therapy can damage healthy tissues surrounding the tumor site, causing adverse effects. Targeted therapy aims to inhibit specific molecular pathways in cancer cells but can be limited by the development of resistance mechanisms. Immunotherapy harnesses the body’s immune system to fight cancer but may not be effective for all types of cancers.

#### Bacterial nanotechnology in cancer therapy

In contrast, bacterial nanotechnology offers a novel paradigm in cancer treatment by leveraging the unique characteristics of bacteria for targeted drug delivery. Bacteria possess inherent abilities that make them attractive candidates for therapeutic interventions. One key advantage is their natural affinity for tumor sites driven by factors like low oxygen levels and inflammation in the tumor microenvironment. This inherent targeting ability allows bacteria to specifically accumulate in tumors, delivering therapeutic agents precisely where needed^3,48–50^.

#### Advantages of bacterial nanotechnology

1. *Targeted drug delivery*: Bacteria can serve as efficient drug delivery vehicles due to their tumor-targeting capabilities, enhancing the localized delivery of therapeutic agents.
2. *Biocompatibility and biodegradability*: Certain bacterial strains are biocompatible and can be safety degraded by the body after delivering their cargo, reducing potential risks.
3. *Enhanced permeability and retention effect*: Nanoparticles carried by bacteria benefit from enhanced permeability and retention within tumors, improving drug efficacy.
4. *Overcoming multidrug resistance*: Bacterial nanotechnology has shown promise in overcoming multidrug resistance mechanisms through innovative delivery strategies.

#### Limitations of bacterial nanotechnology

1. *Safety concerns*: Genetically modified bacteria raise safety concerns related to uncontrolled growth, potential spread beyond tumor sites, and the risk of mutations or horizontal gene transfer^51^.
2. *Limited clinical translation*: Despite promising preclinical studies, the clinical translation of bacterial nanotechnology in cancer therapy remains limited, with few approved nanodrugs currently available^52^.
3. *Immunotoxicity*: The immune response triggered by bacteria-mediated therapies needs careful evaluation to ensure safety and efficacy^30,53,54^.
4. *Complexity of design*: Designing effective bacterial nanocarriers requires a deep understanding of both bacterial biology and cancer pathophysiology^55^.

#### Niche of bacterial nanotechnology

Bacterial nanotechnology occupies a unique niche in cancer therapy by offering a targeted and precise approach to drug delivery that addresses some of the limitations of current standard therapies. By harnessing bacteria’s natural abilities for tumor targeting and drug delivery, researchers aim to enhance treatment efficacy while minimizing systemic toxicity associated with traditional treatments. The potential synergy between bacterial nanotechnology and existing therapies opens up new avenues for combination treatments that could overcome multidrug resistance mechanisms and improve patient outcomes. In conclusion, while bacterial nanotechnology holds great promise for revolutionizing cancer therapy through targeted drug delivery and innovative treatment strategies, several challenges need to be addressed to ensure its safe and effective clinical translation. By carefully navigating these challenges and leveraging the unique advantages of bacterial nanotechnology, researchers can pave the way for more personalized and efficient cancer treatments that offer improved outcomes for patients^56–58^.

### Immune-stimulating hybrid bacterial nano-systems

#### Bacterial toxins

Bacterial toxins are potent nano-sized proteins able to hinder cellular proliferation or alter the cellular machinery that regulates apoptosis, differentiation, and proliferation. When juxtaposed with conventional antitumor therapy, bacterial toxins improved the therapeutic outcome and limited deleterious side effects. for antitumor objectives evinced a reduction in deleterious consequences. Numerous investigations have attested to the effectiveness of *botulinum* neurotoxins (BoNT) in both in vivo and in vitro settings for malignant neoplasms and neoplastic cell populations^59,60^. In this context, BoNTs can inhibit the growth and proliferation of various cancer cells by interfering with a protein crucial for their survival (SV2) and inducing programmed cell death (apoptosis). Additionally, BoNTs can disrupt tumor blood vessels, hindering nutrient supply and potentially enhancing the effectiveness of other cancer therapies. On the immune front, BoNTs might stimulate T cells and natural killer cells within the tumor, bolstering the body’s anti-tumor response. However, a major challenge lies in the immune system potentially developing antibodies against BoNTs with repeated use, reducing their effectiveness. However, several strategies like smaller protein fragments or engineered variants to minimize this immunogenicity and improve the long-term viability of BoNT-based cancer therapies^59,60^. According to a study, the localized administration of BoNT-A into fibrosarcomas and hepato-carcinomas resulted in a noteworthy improvement in tumor Oxygen transport and blood flow. This, in turn, led to an increase in the efficacy of tumor chemotherapy and radiotherapy^61^. The acute cytotoxic effect of *C. perfringens* enterotoxin (CPE), synthesized by *C. perfringens* type A strain, has been observed to induce necrosis of tumors and tumor growth inhibition in pancreatic cancer xenografts expressing claudin-4^62^. While CPE can activate pro-survival pathways such as Erk/Wnt in cancer cells subjected to stress, it may concurrently inhibit their migratory and invasive capabilities. Moreover, CPE treatment elicits an upregulation of genes associated with cell survival (BCL-2, IL-6, IL-8) in stressed cancer cells. On the immunological front, CPE interacts with the innate immune system via TLR3, potentially influencing the tumor microenvironment and eliciting an anti-tumor response from the body. This immune modulation underscores the potential utility of CPE in cancer immunotherapy, although further investigation is warranted to comprehensively elucidate its benefits and potential drawbacks^62^. In addition, it has been observed that diphtheria toxin (DT) can induce apoptosis and trigger cancer cell death by impeding the process of protein synthesis^63,64^. In general, bacterial toxins are antineoplastic agents that can selectively target receptors or pathways implicated in the genesis and advancement of malignancies. Nevertheless, it is imperative to acknowledge the existence of certain limitations that are associated to bacterial toxins such as their immunogenicity, toxicity, stability, and delivery mechanisms. It is essential to undertake additional investigations and engage in the process of optimization in order to enhance the safety and efficacy of bacterial toxins used in the realm of cancer therapy. Immunotoxins are hybrid molecules that combine the specificity of antibodies or ligands with the cytotoxicity of bacterial or plant toxins. They are designed to selectively target and kill cancer cells that express specific antigens or receptors, while sparing normal tissues.

Immunotoxins are highly efficacious agents employed in cancer treatment, exhibiting a discerning affinity for antigens specifically expressed on the exterior of malignant cells. The protein known as *Pseudomonas* exotoxin A (PE) is synthesized by *P. aeruginosa* and is highly toxic. It functions by catalytically ribosylating EF-2, thereby impeding protein synthesis and ultimately resulting in cell lysis. Immunotoxins, commonly synthesized utilizing PE38, exhibit a proclivity for diverse neoplastic masses via the exchange of fusion antibodies or receptor ligands^65^. The IL13-PE38, which is essentially a truncated PE38 protein fused with interleukin 13, has been observed to elicit a direct antitumor cytotoxicity. Additionally, it has been noted to indirectly stimulate a CD8^+^ T cell immune response in the host organism^66^. Other investigations conducted on preclinical models indicate that anti-mesothelin immunotoxins, such as SS1P, elicit antitumor immunity by augmenting the extracellular release of ATP and surface calreticulin expression, thereby facilitating immunogenic cell death and rendering tumors more susceptible to anti-CTLA-4-based treatment^66,67^. Incorporating immunotoxins into a multifaceted therapeutic approach or coupling them with innovative pharmaceutical administration techniques has the potential to enhance their oncolytic efficacy. These innovations underscore the vast capacity of immunotoxins to transform the landscape of cancer treatment methodologies and furnish a compelling justification for continued investigation and enhancement of these agents as constituents of comprehensive therapeutic regimens.

#### Bacteria-Derived Outer Membrane-Based Nano-platforms

Bacterial outer membrane vesicles (OMVs) are produced by Gram-negative bacteria. These nano-vesicles have a lipid bilayer structure that is nano-sized and contains diverse immune-activating components such as virulence factors, enzymes, bacteria-specific antigens, and pathogen-associated molecular patterns (PAMPs)^68^. OMVs have demonstrated their potential as immunotherapeutic agents by efficiently triggering a sustained anti-cancer immune reaction that eradicates established tumors with minimal adverse effects^68^. By administering bacterial OMVs independently, research has shown that they can concentrate in tumor tissue, generate antitumor cytokines IFN-γ and CXCL10 in the TME, and induce antitumor responses^69^. In this regard, the utilization of bacterial extracellular vesicles as an innovative anti-cancer therapeutic approach exhibits significant potential for cancer immunotherapy in the future.

Gram-negative bacteria naturally secrete OMVs with a 20–400 nm size range. Vesicles involve several biological processes, such as horizontal gene transfer, metabolite export, and cell-to-cell communication. OMVs are a type of synthetic nano-vector that stands out from others of similar size due to their inherent biocompatibility, substantial drug-loading capacity, exceptional physicochemical stability, and distinctive biological structure and function that enables communication with cells^70^. The variability of OMVs’ structure and function is contingent upon the bacterial species. Certain types of OMVs possess inherent targeting capabilities and can undergo internalization via endocytosis. *E. coli*-derived OMVs have demonstrated the ability to target and infiltrate melanoma spheroids selectively^71^. Additionally, these OMVs can penetrate the stratum corneum and accumulate in the dermis^72^. OMVs derived from *Salmonella* and *Shigella* possess adhesion molecules that facilitate site-specific delivery systems for colon cancer without requiring any alteration^73^. Furthermore, outer membrane vesicles (OMVs) derived from *Salmonella* and *Shigella* exhibit immunogenic properties capable of activating both innate and adaptive immune responses. Constituents within OMVs, including lipopolysaccharides (LPS) and outer membrane proteins, have the capacity to induce the secretion of pro-inflammatory cytokines and facilitate the activation of immune cells such as macrophages, dendritic cells, and T cells^73^. The immune-stimulatory nature of *Salmonella* and *Shigella* OMVs suggests their potential use as adjuvants or delivery vehicles in cancer immunotherapy, enhancing the body’s anti-tumor immune responses. Prior investigations have indicated that bacterial OMVs have the capacity to carry a range of anti-cancer drugs, such as RNA, DNA, Indocyanine Green (ICG), paclitaxel, TNF-related apoptosis-inducing ligand (TRAIL), and to facilitate thermo-therapy, chemotherapy, and immunotherapy^19^.

OMVs have demonstrated considerable potential as nano-carriers within the realm of targeted drug delivery when subjected to bioengineering techniques. Some investigations involved the fabrication of a recombinant protein OMV, which encompasses a particular composition. Bioengineered OMVs exhibit auspicious prospects as a nano-carrier in the realm of targeted drug delivery. In a particular investigation, scholars fabricated a hybrid protein termed OMV containing cytolysin A (ClyA) and possesses the ability to be infused with small interfering RNA (siRNA) for expression in *E. coli*. ClyA is a cytotoxin engineered with a high degree of specificity to target neoplastic cells selectively^70,74^. The OMVs were subjected to electroporation with siRNA constructs designed to target kinesin spindle protein^70^. The in vitro results of this system demonstrated favorable cytotoxicity, while in vivo, it effectively inhibited tumor growth^70^. A recent investigation has demonstrated that OMVs that are adorned with Epidermal Growth Factor Receptor Variant III (EGFR vIII) and B16-M30 have elicited elevated levels of anti-EGFR vIII antibody titers, M30-specific T cells, and the infiltration of CD4^+^ and CD8^+^ T cells at the tumor site^50^. According to a recent study, the surface expression of a PD-1 ectodomain on bioengineered OMVs has been found to provide protection to T cells against PD-L1-mediated inhibition and apoptosis, thereby leading to the infiltration of T cells into tumors^75^. The administration of this therapeutic intervention resulted in a comprehensive regulation of the TME, thereby inducing a notable enhancement in the effectiveness of the antitumor response^75^. A recent investigation has demonstrated that bioengineered OMVs enveloped with nanomedicines have the ability to directly engage with immune cells, thereby modulating tumor immunity and forestalling metastasis. The efficacy of this technology was further demonstrated in conjunction with immunomodulatory agents, as evidenced by an 81% success rate^76^. Furthermore, the utilization of OMVs that have been functionalized with nanomicelles containing tegafur has resulted in the activation of an immune system reaction that is effective against cancer^77^. Additionally, these OMVs have been observed to sensitize cancer cells to cytotoxic T lymphocytes. The synergistic interplay between the immunomodulatory and chemotherapeutic effects of tegafur culminated in the eradication of cancerous cells^77^.

**Fig. 6:**
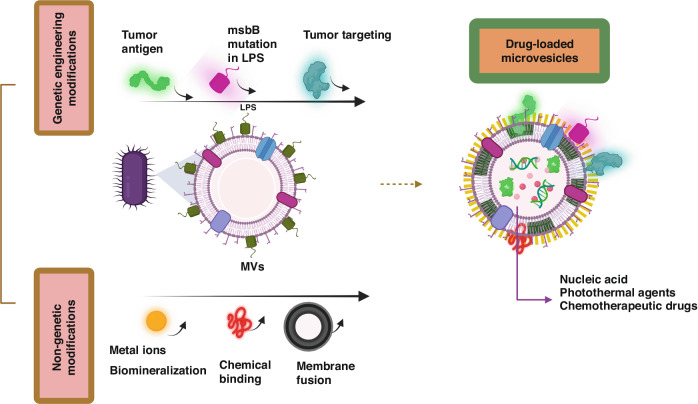
Visualizing drug-loaded microvesicles engineered through non-genetic and genetic approaches. Genetic manipulation has been utilized to decrease pathogenicity, improve targeting ability towards tumors, and enhance anti-tumor immune response. Non-genetic engineering modifications have also been employed, such as biomineralization, chemical bonding, and membrane fusion. The resulting functionalized microvesicles (MVs) have the capability to carry various drugs. Created with BioRender.com

It has been observed that microvesicles (MV) derivate of bacteria consists of several immunostimulatory elements of bacterial origins, such as LPS, lipoproteins, DNA, RNA, and outer membrane proteins^78^. This discovery prompted extensive research into the potential benefits of treating tumors with MVs alone^78^. However, when administered via intravenous injection, severe systemic inflammatory reactions and quick elimination were observed in mice^79^. To broaden the application of MVs in anticancer immunotherapies, various approaches have been examined to develop improved drug delivery carriers. This section is dedicated to discussing the development of techniques for utilizing bacterial MVs as drug carriers to attain improved anticancer results. This involves modifying biosynthesis through genetic means to produce intrinsic species or attaching extrinsic species to the membrane surface, as illustrated in Fig. 6.

**Fig. 7:**
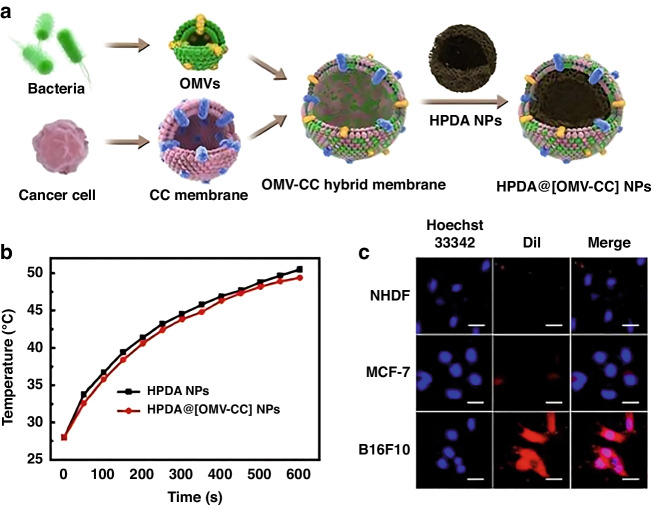
Hybrid Membrane Formation and Properties of HPDA@ [OMV-CC] NPs. **a** Diagram of the hybrid membrane formed by combining OMV and cancer cell (CC) membranes and the resulting HPDA NPs coated with the hybrid membrane to produce HPDA@ [OMV-CC] NPs. **b** Temperature increase of HPDA@[OMV-CC] and HPDA NPs NPs (100 μg/mL). **c** CLSM image of MCF-7 cells, B16-F10 cells, and NHDF cells, cultured with DiI-dyed HPDA@[OMV-CC] NPs and stained with Hoechst 33342. Adapted with permission from original source^81^. Copyright 2020 American Chemical Society

The unique features of MV surfaces provide opportunities for modifying them to enhance their safety and effectiveness as a therapeutic tool against cancer. One strategy that has been explored involves “shielding” the MVs by enclosing them in highly biocompatible nanomaterials, such as those formed through membrane fusion, chemical bonding, or biomineralization^19^. The utilization of chemical modifications for the alteration of MVs has been comparatively less explored in comparison to alternative techniques^19^. One study involved synthesizing a biocompatible calcium phosphate layer on OMVs to envelope them. The CaP shells dissolved in the mildly lower pH of TME, releasing the MVs to activate immune responses against tumors^80^. Qing et al. incorporated folic acid into the calcium phosphate (CaP) shells to augment tumor targeting. Folic acid, a non-immunogenic ligand, was chosen due to its ability to selectively bind to the folate receptor, which is commonly overexpressed on the surface of numerous cancer cells. They utilized membrane fusion technology, which depends on the fact that many cell membranes are structurally similar, to achieve the fusion of two natural bio-membranes^80^. In parallel, Wang et al. utilized a fusion technique to combine bacterial MVs with the membrane of malignant cells, resulting in a hybrid membrane^81^. This hybrid membrane exhibited both the homing ability of the cancer cell membrane and the immunostimulatory capacity of bacterial MVs. The application of this technique proved to be effective in treating melanoma. This strategy has the potential to be adapted for immunotherapy and other treatments for different types of cancer by fusing bacterial MVs with various cancer cell membranes and incorporating different therapeutic agents inside the MVs (Fig. 7)^81^. In other word, the hybrid membrane merges the immunostimulatory potential of bacterial MVs with that of the cancer cell membrane. This combination yields a membrane capable of eliciting immune responses, thereby potentially bolstering anti-tumor immunity within the tumor microenvironment. In addition, owing to the similar lipid structure, DSPE-PEG-RGD has been integrated into the lipid bilayer of microvesicles (MVs) using extrusion technique, with the lipid head of DSPE, in order to augment the targeting proficiency of MVs. This method has been applied to cover MVs onto nano-micelles loaded with Tegafur, leading to enhanced cancer immunotherapy through both immunomodulatory and chemotherapeutic effects^82^.

### Hybrid bacterial nano-systems as chemotherapeutic delivery vehicles

Several nanomaterials have been studied for the purpose of creating a hybrid delivery system for bacteria together with chemotherapeutic agents. This system aims to enhance the efficacy of antitumor therapy. Various types of drug delivery systems, including micelles, liposomes, and others, have demonstrated noteworthy advantages in drug delivery and loading^83,84^. Polyethylene glycol-modified nanoparticles are considered potential candidates owing to their biocompatibility and flexibility. On the other hand, various types of cargo nanomaterials, including alginates, polycaprolactone, cellulose, polystyrene, and chitosan, have been utilized for this purpose^85,86^. The density of attachments, cargo shape, and non-uniform degree of coupling on the bacteria’s surface, specifically the bifacial surface patterning, are factors that can significantly affect the motility and receptiveness of bacteria^87,88^. The bacteria’s capacity to target tumors may also be influenced by these factors. Understanding these factors influencing bacterial motility and receptiveness through surface modifications is crucial for optimizing engineered bacteria in various applications, such as bioremediation, targeted drug delivery, and biosensing. Overly dense modifications can compromise bacterial viability, and unintended cargo interactions can lead to undesirable consequences. Therefore, a delicate balance needs to be achieved when engineering bacterial surfaces to achieve the desired functionalities. This optimization is particularly important when considering propulsion, as evidenced by previous research documenting how nanomaterials affected the propulsion velocities of the modified hybrids, with a range of 0.5 m/s to 30 m/s reported. This wide range of velocities highlights the significant impact surface modifications can have on bacterial motility^89,90^.

Despite significant advancements in nanomedicine delivery for cancer treatment, extracellular matrix obstruction and interstitial fluid pressure pose significant barriers to their accumulation in cancerous tissues. Hybrid delivery systems that leverage active targeting through bacterial migration and the superior drug-loading performance achieved by nanoparticles have yielded impressive results in targeted therapeutic agent delivery^58^. For example, Suh et al. developed a hybrid system based on bacteria-enabled autonomous delivery^46^. Their study utilized a streptavidin-biotin interaction to bind PLGA nanoparticles to the *S. typhimurium* VNP20009 bacterium, which did not compromise bacterial targeting or tumor penetration^46^. This conjugation resulted in a 100-fold higher concentration of PLGA nanoparticles in tumors than passively diffusing nanoparticles. This significant increase in tumor accumulation is attributed to the bacteria’s inherent tumor-targeting properties, which allowed the nanoparticle-loaded bacteria to selectively localize and accumulate in the tumor microenvironment^46^. Subsequent studies should focus on assessing the effectiveness of this platform with regard to therapeutic results.

Another innovative hybrid bacterial nano-system developed by Luo et al. implements high-intensity focused ultrasound (HIFU) treatment for oncologic intervention. Their investigation involved conjugating PLGA nanoparticles loaded with perfluorohexane (PHF) onto *Bifidobacterium*, demonstrating strong tumor-targeting abilities, improved diagnostic efficiency, and enhanced therapeutic effectiveness^18^.

Bacteria have the ability to increase the concentration of nanoparticles at cancerous sites and facilitate the transportation of cargo to their specific subcellular destinations. The drug-carrying capability of bacterial carriers is enhanced by the presence of nanoparticles. The utilization of bacteria-nanoparticle hybrid transport systems presents a unique benefit due to their proficient ability to target cancer, effective capacity for loading drugs, and established delivery to subcellular regions^91^. Understanding the effect of nanoparticle components on the bacteria’s capacity to target tumors is essential. In general, the tumor-targeting capability of nanoparticles decreases as the amount of loaded nanoparticles decreases^90^. It is essential to examine the impact of the conjugation method utilized for connecting nanoparticles and bacteria on their capacity to transport medications to their designated objectives. Various conjugation methods, including electrostatic attachment, physical attachment, and antigen-antibody-specific interaction, can lead to different robustness levels in the biological milieu. This can affect their capacity to deliver drugs accurately to their intended destination^91^.

The development of bio-hybrid nano-robotic systems faces several challenges that must be overcome for effective drug delivery to tumor sites. Achieving precise and controlled drug release is essential for effectively targeting the tumor micro-environment. These conditions may include lower pH levels, high matrix metallase and glucuronidase expression. as well as exposure to ultrasound stimuli or light^92^. The incorporation of intelligent nanoparticles into the hybrid system may enhance drug release efficacy. Secondly, it is imperative to prevent uncontrolled bacterial proliferation in the body, which could trigger an autoimmune response leading to severe adverse reactions and even fatal consequences. Although weakened bacteria and avirulent strains have been explored, the risk of bacterial surface components such as lipopolysaccharides still remains. Synthetic biology presents various strategies to address this issue, including the design of auxotrophic bacteria or constructing suicide circuits within bacterial cells, in compliance with regulatory requirements^93^. Notably Lim et al. created SimCells and mini-SimCells using *Pseudomonas putida*, *E. coli*, and *Ralstonia eutropha*. These cells were designed to be chromosome-free and could potentially be used to regulate bacterial growth within the human body^94^. In this regard, the SimCells and mini-SimCells are engineered to display nanobodies on their surface that can specifically bind to carcinoembryonic antigen (CEA), a common biomarker found on colorectal cancer cells. This targeted binding enables the selective accumulation and delivery of therapeutic payloads directly to the CEA-expressing cancer cells, inducing targeted cell death^58^. The incorporation of additional therapeutic agents, such as pore-forming proteins or chemotherapeutics, can further enhance the cancer-killing effects of these bacterial constructs. On the immune system front, the SimCells and mini-SimCells possess the potential to stimulate anti-tumor immune responses by incorporating immunostimulatory components, such as cytokines or adjuvants, within their engineered structure^58^. Additionally, their non-replicating and highly controllable nature may allow them to evade or minimize unwanted immune responses, thereby improving their targeted delivery and therapeutic efficacy. Overall, the versatility of the SimCells and mini-SimCells, with their ability to selectively target cancer cells and potentially modulate the immune system, represents a promising approach for developing more effective and personalized cancer treatment strategies^58,94^^.^ In the future, safer and protein-expressing bacterial cells could be engineered and combined with nanomaterials to achieve the ideal drug delivery system.

#### Very small-size proteoliposomes (VSSPs)

VSSPs are generated through the application of an anionic detergent to bacterial OMVs, with the concomitant inclusion of mono-salic acid dihexosyl ganglioside (GM3) into the vesicular structure^50,95,96^. The GM3 molecule, a constituent of the plasma membrane in mammalian cells, has been identified as a promising target for cancer immunotherapy. The utilization of VSSPs has the potential to improve effectiveness in eliciting immune recognition directed towards gangliosides. The N-glycosylated variant of GM3, known as NGcGM3, has been detected on neoplastic cells and has been identified as a potential immunotherapeutic target for certain malignancies in humans, including but not limited to metastatic melanoma and breast cancer. The NGcGM3 ganglioside vaccine has been formulated as a precision medicine for cancer treatment. It has been demonstrated that the NGcGM3/VSSPs vaccine is both safe and immunogenic in a subset of patients with metastatic melanoma^97,98^. Therefore, by incorporating this ganglioside into the VSSP structure, the system can potentially improve the effectiveness of eliciting an immune response directed towards the cancer cells expressing this tumor-specific antigen. The demonstrated safety and immunogenicity of the NGcGM3/VSSPs vaccine in metastatic melanoma patients further highlights the potential of this approach in cancer immunotherapy.

Another study demonstrated that VSSPs could enhance the expression of CD86 through their interaction with Toll-Like Receptor 2 (TLR2) situated on the surface of Antigen-Presenting Cells (APCs). This phenomenon renders them a potent adjuvant that can trigger the activation of Dendritic Cells (DCs) in both human and murine models. The initiation of this process results in the generation of TNF-α, IFN-γ, IL-12, IL-10, and IL-6^95^. It is of significance to note that the combination of VSSPs and anti-PD-1 therapy resulted in a notable extension of the survival rate in mice that were carrying tumors. Furthermore, the in vitro administration of VSSP treatment has been observed to induce M1-like polarization in tumor-associated macrophages (TAMs) among patients afflicted with metastatic ovarian cancer, thereby mitigating their inhibitory phenotype to a certain degree^99^. The utilization of VSSP therapy in isolation or conjunction with other therapeutic modalities exhibits auspicious potential for forthcoming biomedical implementations.

#### Bacteria as nano-carrier of therapeutic agents

The S-layer, a crucial component of bacteria, is highly useful in creating bionic nanoparticles. A type of lipid nanoparticle called solid lipid NPs, which are tiny lipid-based particles, is commonly employed to transport drugs not soluble in water^100,101^. The procedure of coupling the S-layer with lipid-based nanoparticles can be achieved through either physical or chemical bonding methodologies. There exist two distinct classifications of lipid membranes that are enveloped by S-layer proteins (Slp), namely emulsomes, and liposomes. The utilization of S-layer coated emulsion has been observed to be efficacious in transporting drugs that exhibit either hydrophilic or hydrophobic properties^100^.

The findings of in vitro investigations have substantiated that a particular emulsion is capable of being assimilated by hepatocellular carcinoma cells in humans at a diverse spectrum of concentrations (50 μg/mL) while being non-toxic^102^. An alternative approach entails the alteration of liposomes utilizing S-layer proteins, which possess the ability to encapsulate compounds of both lipophilic and hydrophilic nature. The utilization of S-layer coated liposomes has exhibited superior hemodynamic persistence and robustness in comparison to emulsions. This phenomenon might be due to the fact that Slp coating on the liposomes serves as a “protective suit” that can block biological interactions, avoiding recognition by opsonins and achieving extended circulation in the body. This reduced biological interaction helps to prolong the blood circulation time of the Slp-coated liposomes, facilitating their accumulation in the tumor tissue via the enhanced permeability and retention (EPR) effect^102^. Notably, liposomes coated with sbPA-S demonstrate noteworthy cellular uptake in the HeLa human cancer cell line, particularly when possessing a positive charge^103^. It is noteworthy that recent studies have demonstrated the capacity of S-layer proteins, which act as natural immunoadjuvants, to assemble on the surface of S-CM-HPAD NPs. This assembly has been found to properly protect antigens and enhance anti-tumor immunity by stimulating T cell proliferation and cytokine secretion as well as interacting with cancer cells by enhancing their cellular uptake and enabling targeted drug delivery^104^.

The utilization of nanopatterned S-layer fusion proteins that exhibit diverse functionalities has the potential to enhance the application of emulsion and liposomes in the domain of nanomedicine. This particular methodology may improve the efficacy of drug delivery and targeting capabilities^105^.

#### Bacterial biopolymers

Numerous types of bacteria possess the ability to transform diverse carbon sources into different types of biopolymers, which can subsequently be employed to generate nanoparticles (NPs) suitable for a drug delivery system (DDS). Bacterial polymers, comprising of bacterial polysaccharides like xanthan gum, gellan gum, and hyaluronic acid (HA), along with fructans like levan, exhibit potential as drug encapsulation agents^106^.

Hyaluronic acid (HA) is a type of linear-chain polysaccharide that consists of alternating sequences of D-glucuronic acid and N-acetyl-D-glucosamine units. These units are linked together by a (1 → 4) glycosidic bond. The protein CD44, which is located on the surface of cells and has the ability to bind to hyaluronic acid (HA), has been found to be expressed at higher levels in multiple types of cancer cells^107^. The aforementioned characteristic renders HA a viable candidate as a ligand for precise administration of anticancer medications. The synthesis of nanomaterials was achieved through the combination of amphiphilic HA and hydrophobic bile acids. It was observed that the SCC7 cells exhibited the capability of internalizing HA nanoparticles through CD44 receptor-mediated endocytosis^108^.

A nanoconjugate consisting of hyaluronic acid (HA) and paclitaxel has been proposed as a novel and valuable nanocarrier^109^. For the development of a drug delivery system (DDS) targeting the liver, thiolated HA was chemically linked to gold nanoparticles and then bound to IFN-α through electrostatic and hydrophobic interactions^110^. Graphene oxide-HA (GO-HA) has been successfully employed in cancer treatment by utilizing CD44-mediated endocytosis, and it also demonstrates pH-dependent release of epirubicin in acidic lysosomes^111^. Additionally, another study revealed that PEGylation of HA reduces cellular uptake, which prevents excessive accumulation in the liver following systemic administration^112^.

Epigallocatechin gallate is a compound present in green tea that has anticancer and antioxidant properties. A ternary system composed of hyaluronic acid (HA), epigallocatechin gallate, and linear polyethylenimine was developed for delivering proteins (such as granzyme B and lysozyme) to cancer cells in a targeted manner^113^. In this regard, the delivery of cytotoxic proteins, such as granzyme B (GrB) and lysozyme, has emerged as a promising approach in cancer treatment. Granzyme B, a serine protease naturally released by cytotoxic T lymphocytes (CTLs) to induce apoptosis in target cells, has been the focus of extensive research^113^. Novel GrB nanoparticle delivery systems have been developed to mimic the functionality of CTLs, allowing for the direct delivery of GrB to cancer cells and the induction of efficient cell death. The cationic nature of lysozyme, another protein with therapeutic potential, has been exploited to facilitate the binding and delivery of granzyme A (GA) to target cells. This synergistic approach leverages the targeting capabilities of lysozyme to enhance the internalization of granzymes into cancer cells. Importantly, granzymes, including GrB and GA, have been found to enter the mitochondria through a non-canonical import pathway involving the Sam50, Tim22, and mtHsp70 proteins, which is crucial for their ability to induce effective cell death in cancer cells^114^. Furthermore, the dual-targeting capability of GrB, against both cancer cells and bacteria, has been observed, with GrB demonstrating the ability to target and kill bacteria by entering their cytosol, thereby attenuating bacterial virulence. The versatility of these cytotoxic protein delivery systems, combined with their targeted and mitochondrial-localized mechanisms of action, highlights their significant potential as innovative cancer therapeutics^115^. In another combinatory therapy, it was shown that the use of IDO inhibitor-loaded HA-graphene oxide (GO) nanosheets with mesothelin chimeric antigen receptor T (CAR-T) cells could increase cytokine secretion and enhance the cytotoxic activity of the CAR-T cells. This led to an increase in the expression of IFN-γ and IL-2 and a decrease in PD-1 and TIM3 expression^116^.

Thanks to the genetic systems and engineered metabolic pathway technologies, bacteria are a promising choice for producing microorganisms for drug development. Biopolymers offer a range of design possibilities for drug development, but additional research is needed to enhance their effectiveness and targeting towards tumors. Table 1 provides examples of drug delivery systems derived from various bacteria for cancer therapy strategies.

**Table 1:** Cancer treatment with bacteria as drug carriers

Treatment category	Treatment approach	Approaches	Cancer category	Efficiency	References
Disease-causing bacteria	*Bacillus Calmette Guerin* (BCG)	Stimulated cancer-targeted T-cell response	Urothelial carcinoma	boosted killing capacity of neoplasm-specific CD4 + T cells, upregulated IFN-γ expression, stimulates the IFN-γ receptor on malignancy cells, triggering malignancy-restricted immunity	^48^
	Weakened S. typhimurium	Transfer of bacterial plasmid carrying endostatin and siRNA targeting Stat3 genes into eukaryotic cells	Primary hepatic cancer	Suppressed neovascularization, Suppression of tumor invasion and metastasis	^193^
		Oral administration of weakened bacteria carrying a plasmid that expresses siRNA-Stat3 in eukaryotic cells	Neuroblastic tumor	Increased activity of NK cells and function of T cells	^177^
		Host IDO expression is silenced in the VNP20009 strain of *S. typhimurium* bacteria	Non-Hodgkin lymphoma	Caused substantial tumor penetration and enhanced cell death within the tumor	^198^
		Using a weakened mutant strain of *S. typhimurium* that does not have the ZnuABC transporter to treat breast cancer	Breast cancer	Slowed down tumor progression and prolonged mice survival by killing cancer cells and stimulating immune reaction	^194^
	*L. monocytogenes*	Weakened *Listeria* invaded MDSCs in the cancer site	Breast cancer	Enhanced NK cell and T-cell activities and changed immune-inhibiting TME to immune-stimulating	^199^
		Using weakened Δ act A/Δ inlB strain of *L. monocytogenes* to treat a aggressive ovarian cancer model	Ovarian cancer	Changed TAMs from M2 to M1 type and prolonged mice life span	^53^
		Used weakened strain of AH1 producing *L. monocytogenes* in a mouse model of metastasic colorectal cancer	Metastatic colorectal cancer	Triggered strong memory and primary T-cell reactions and prevented mice from tumor recurrence	^200^
		To treat cervical cancer in mice with immunotherapy	Cervical cancer	Raised levels of IL-17 and IL-17-producing IL T cells in the tumor	^201^
Nonpathogenic bacteria	*Lactobacillus rhamnosus* GG (LGG)	Received either active or freeze-dried LGG through bladder infusion	Orthotopic bladder cancer	Employed huge quantity of and macrophages neutrophils to the cancer site and prohibited tumor progression in bladder cancer	^202^
	*Bifidobacteria*	The combined therapy with anti-PD-L1 and *Bifidobacteria*	Melanoma	Boosted dendritic cell activity and stimulated antigen specific CD8^+^ T cells and eradicated the tumor progression	^203^
	Non-pathogenic *E. coli* Nissle	Intra-tumoral injection of *E. coli* producing anti-CD47 nanobody	Lymphoma, breast cancer, and melanoma	Enhanced stimulation of T cells within the tumor and triggered systemic immune responses to tumor antigen	^204^
		SYNB1891 was recruited for expression of the STING-agonist cyclic diAMP to selective stimulation of phagocytic antigen-presenting cells (APCs) in the TME	Colon carcinoma lymphoma, breast cancer, and melanoma,	Produced effective immune responses against the tumor	^205^
Bacterial Toxins	*Pseudomonas* exotoxin A (PE)	Intratumoral injection of IL13-PE38 into mice bearing D5 melanoma tumors	Melanoma	Caused tumor regression and generated adaptive immune responses preventing tumor recurrence	^206^
		The effect of combined therapy with SS1P injected locally and anti-CTLA-4 administered intraperitoneally	Mesothelioma	Enhanced anti-cancer response from combining local PE immunotoxins and systemic anti CTLA-4 immune checkpoint inhibitors	^207^
Bacterial OMVs	*E. coli*	Cancerous tissue is targeted and accumulated by OMVs injected systemically	Colorectal adenocarcinoma, melanoma and breast cancer	Triggered the release of anti-cancer cytokines CXCL10 and IFN-γ	^69^
Bacterial spores	*C. novyi*-NT	*C. novyi*-NT spores administered Intravenously	endogenous neoplasia	Enhanced the production of TNF-α in response to LPS, IL-10 in response to LTA and the function of NK cell-like cells	^208^
		Delivering *C. novyi*-NT spores to the recipients	Renal cell malignancy and colorectal adenocarcinoma	Enhanced the innate immunity and the CD8^+^ T cells that target cancer to trigger lasting immune system modifications	^209^
		Delivered attenuated *C. novyi*-NT into the tumor that did not respond to treatment		Caused local tumor elimination and anti-tumor immune reactions and activated inflammatory	^210^

#### Encapsulation of compounds by bacterial ghosts (BGs)

Bacterial ghosts, or BGs, are bacterial cell envelopes that have undergone a process of content extraction facilitated by a protein E-mediated channel in the cell membrane. This channel is encoded by the phage PhiX174 gene in *E. coli*^51^. Bacterial ghosts exhibit a remarkable capacity for bearing substantial loads and can serve as a proficient vehicle for transporting vaccines and medicinal agents. Biliary glycoproteins exhibit a remarkable degree of specificity in their ability to target particular tissues selectively and have demonstrated notable efficacy in being internalized by cells of colon cancer, leukemia, and melanoma^51^. Recent research has demonstrated that phenolic compounds, such as resveratrol, have the ability to bind with BGs. This binding mechanism facilitates the delivery of BGs to macrophages in a targeted manner, thereby mitigating the production of NO induced by BGs, without any adverse cytotoxic effects^117^. The augmentation of the resveratrol effect through intracellular transport by BGs facilitated the presence of nuclear and intracellular receptors for resveratrol that have the potential to enhance the biological signal produced by this compound. The utilization of Ciprofloxacin-loaded BGs (BG@Cip) has been observed to stimulate macrophages into the secretion of cytokines, including but not limited to TNF-α interleukin-6 and (IL-6)^118^. Overall, the versatility and potential applications of BGs in drug delivery, tissue engineering, and immunotherapy, make them promising candidates for developing novel therapeutic strategies and improving clinical outcomes. Further research is needed to fully understand the mechanisms underlying these effects and optimize the design and formulation of BGs for specific therapeutic applications.

### Photocatalytic therapy using hybrid bacterial nano-systems

**Fig. 8:**
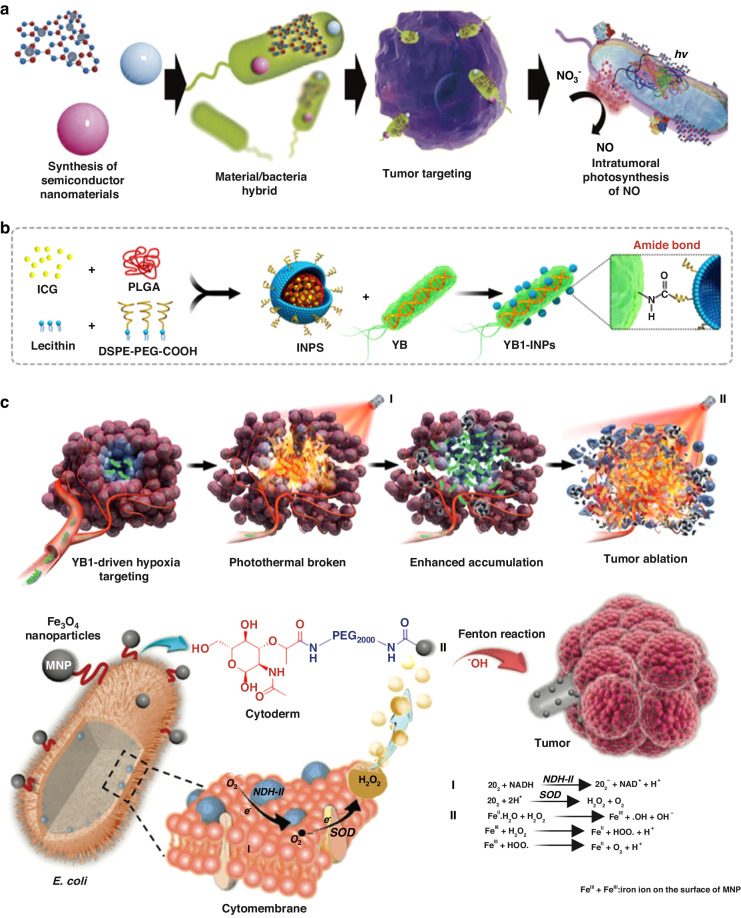
Nanobacterial Therapies for Tumor Treatment: Mechanisms and Applications. **a** How bacteria can be used to treat tumors with light and chemicals. **b** A diagram shows how light can activate bacteria to kill cancer cells. Adapted from^120^, 2018, Springer Nature. **b** How YB1–INPs are made and how they work. YB1–INPs are nanoparticles that stick to a type of bacteria called YB1. They can target tumor cells with low oxygen levels and heat them up with near-infrared (NIR) light. This breaks down the tumor cells and attracts more bacteria to the tumor site. This leads to more tumor destruction and prevents tumor regrowth. Adapted from ref. ^124^, 2019, Elsevier. **c** A diagram showing how bacteria can act as a bioreactor that produces chemicals that react with iron and oxygen to damage tumor cells. Adapted from ref. ^212^, 2019, WILEY-VCH Verlag GmbH & Co. KGaA, Weinheim

Nanostructures possess the capability to serve not only as mere carriers for pharmaceutical substances, but also as dynamic entities themselves, thereby augmenting the efficacy of bacterial therapy across a diverse range of medical treatments, including enzyme-like therapies. Within this particular segment, we shall engage in a comprehensive discourse regarding a multitude of pioneering therapeutic methodologies that effectively employ hybrid bacterial nano-systems. Photocatalytic therapy encompasses a dual sequential modality wherein photosensitizers are initially concentrated at the desired tissue site, followed by the application of light to trigger the activation of these agents^119^. Zheng et al. have devised a novel approach to bacterial therapy, employing the principles of photo-control. This method involves the synergistic combination of carbon nitride (C_3_N_4_) with the bacterium *E. coli*, facilitated electrostatic attraction^120^. Under the influence of illuminative radiation, the C_3_N_4_ material generated photoelectrons, subsequently infiltrating *E. coli*. This infiltration led to a notable augmentation in the enzymatic reduction process, specifically the conversion of endogenous NO_3_^-^ into the perilous NO molecule (Fig. 8A). The incorporation of this therapeutic approach led to an impressive tumor remission rate of approximately 80% (in contrast to the mere 20% reduction observed with the administration of *E. coli* alone)^120^.

### Photothermal therapy (PTT)

Photothermal therapy (PTT) is a therapeutic modality that harnesses the power of photosensitizers (PSs) to target and eliminate cells specifically. Photothermal therapeutic agents possess the remarkable ability to effectively harness electromagnetic energy, thereby facilitating the conversion of luminous energy into thermal energy. This thermal energy can be strategically employed to eliminate malignant tumor cells with utmost accuracy and precision selectively. Within the realm of targeted drug delivery, bacteria serve as vehicles for the transportation of pharmaceutical substances to effectively reach and engage with solid tumors that are actively proliferating. Once photosensitizers are attached to the bacteria, they may be subjected to the influence of a near-infrared laser in order to incite the photothermal effect. The bacteria harboring PSS exhibited noteworthy efficacy in eradicating neoplastic cells and cohesive neoplasms^54,121^.

The advent of PTT has given rise to the conception of a groundbreaking immunotherapeutic adjuvant strategy known as photothermal CpG nanotherapeutics (PCN). CpG, an oligodeoxy nucleotide composed of cytosine-phosphate-guanine, exhibits the remarkable ability to serve as a Toll-like receptor (TLR) agonist, thereby eliciting the activation of innate immune responses. This, in turn, has the potential to augment the immune system’s specific reaction to vaccines. In the context of PolyCationic Nanoparticles (PCN), the cytosine-guanine dinucleotide motif (CpG) is linked to ovalbumin (OVA), a carrier protein that is frequently employed in vaccine development. This conjugation process is accompanied by the attachment of CpG-OVA complex to gold nanorods, which fulfill the vital role of photothermal conversion agents. The attainment of localized heating is accomplished by intra-tumoral administration of photothermal conversion nanoparticles (PCN) in conjunction with near-infrared (NIR) light radiation^122^. This process induces a hyperthermic condition akin to fever, wherein the temperature reaches 43°C, thereby engendering a conducive immunological milieu within the TME^123^. This mechanism facilitates the immune system’s ability to inhibit the growth of tumors by enhancing the efficacy of CpG-based immunotherapeutic approaches It has also been observed that the utilization of a synergistic approach, wherein immune-assisted nanoparticles employing photothermal therapy (PTT) are combined with immune checkpoint blockade (ICB) therapy, yields enhanced outcomes in terms of the antitumor immune response. PTT, a cancer treatment modality that utilizes light-absorbing agents to generate heat and induce tumor cell death, can trigger a cascade of immunological events that synergize with the mechanisms of ICB therapy^124^. Specifically, PTT can induce immunogenic cell death in tumor cells, leading to the release of tumor-associated antigens and damage-associated molecular patterns (DAMPs)^38^. This release of tumor antigens and DAMPs stimulates the activation and maturation of antigen-presenting cells, such as dendritic cells, priming the adaptive immune system against the tumor. Furthermore, PTT can modulate the tumor microenvironment by increasing the infiltration of cytotoxic T cells and reducing the presence of immunosuppressive cells, such as regulatory T cells and myeloid-derived suppressor cells^38^. This remodeling of the tumor microenvironment can enhance the efficacy of ICB therapy by relieving the immunosuppressive barriers and promoting a more favorable immune landscape for antitumor responses. The combination of PTT and ICB therapy has been shown to have synergistic antitumor effects, leading to improved tumor regression, inhibition of metastases, and prevention of tumor relapse. This complementary approach, where PTT induces immunogenic cell death and modulates the tumor microenvironment, while ICB therapy reinvigorates the adaptive immune response, results in enhanced systemic immunity and the generation of memory T cells that can provide long-term protection against tumor recurrence and metastasis^33,38,125^. The amalgamation of therapeutic modalities has demonstrated a remarkable capacity to impede the metastatic dissemination of tumors and prevent their resurgence subsequent to localized tumor ablation across diverse tumor models. The phenomenon of tumor-specific thrombosis induced by bacteria gives rise to tumors that exhibit a notably darker appearance and possess a heightened capacity for near-infrared (NIR) absorption. This, in turn, culminates in the successful implementation of tumor photothermal ablation, a highly effective therapeutic approach^125^.

**Fig. 9:**
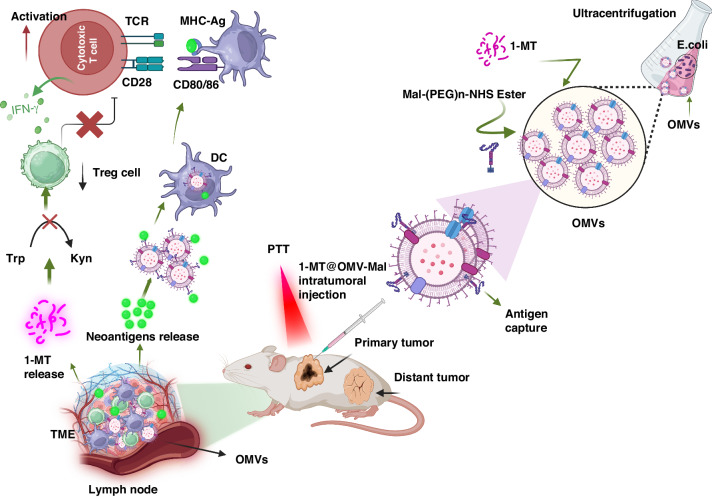
Schematic diagram of preparation of 1-MT@OMV-Mal and its antitumor mechanism. Created with BioRender.com

In a study, Chen et al. used nano-photosensitizers conjugated to a genetically modified and safe *S. typhimurium* strain called YB1 to treat cancer. The nano-photosensitizers, loaded with indocyanine green (ICG) nanoparticles, were conjugated to YB1 using an amide bond (YB1-INPs). After intravenous injection and accumulation in the tumor, the tumor cells were lysed with the first application of near-infrared (NIR) light. This released nutrients that attracted more bacteria to the cancer tissue, allowing for bacterial enrichment. The second NIR irradiation was then incorporated to entirely exterminate the established solid tumor without relapsing (Fig. 9B)^124^.

In another study, the administration of OMVs obtained from *S. typhimurium* via intravenous injection exhibited a notable increase in the levels of antitumor cytokines. Tumors subjected to the administration of OMVs exhibited a noteworthy escalation in the optical absorbance within the intra-tumoral region, specifically in the NIR spectrum. This led to a successful implementation of photothermal ablation, wherein the tumors were effectively eradicated upon exposure to a NIR laser^126^. In a subsequent study, the authors devised a multifaceted in situ vaccine termed 1-MT@OMV-Mal, which effectively harnesses OMVs for capturing antigens from the tumor and modulating the immune system’s response. This strategic intervention aims to bolster the immune-mediated eradication of tumors subsequent to PTT. Within this intricate and cohesive cancer immunotherapy framework, OMVs underwent a process of modification wherein maleimide groups (Mal) were introduced (Fig. 9). This alteration facilitated the binding of the OMVs to tumor antigens that were released subsequent to PTT. The DCs acknowledged and embraced these modified OMVs to stimulate the activation of antigen-specific T cells. Furthermore, it is worth noting that the interior of extracellular vesicles known as OMVs was effectively infused with an inhibitor of indoleamine 2,3-dioxygenase (IDO), specifically 1-methyltryptophan (1-MT). This strategic intervention was undertaken to counteract the immunosuppressive microenvironment orchestrated by regulatory T cells (Tregs). The outcome of this endeavor was nothing short of remarkable, as it yielded significant inhibition effects on both primary and distant tumors^127^. The investigation also delved into the examination of the combined impact of PTT and immunotherapy. This entailed the administration of a blend of N-dihydrogalactose-chitosan (GC) glucosamine polymer, an immune adjuvant, and Indocyanine green (ICG), an FDA-approved photothermal therapy probe. The objective was to eradicate any remaining primary and metastatic tumor cells through near-infrared laser irradiation. The administration of this therapeutic intervention yielded a heightened immune response to tumors and demonstrated sustained efficacy over an extended period^128,129^.

### Chemodynamic therapy (CDT)

Chemodynamic therapy (CDT) is a novel cancer treatment strategy that uses compounds that involve Fenton or Fenton-like reactions to generate hydroxyl radical (·OH) with high cytotoxicity for inducing cancer cell apoptosis. CDT is defined as in-situ treatment and has advantages such as tumor specificity, no need of external stimuli, and low side effects. It also faces challenges such as the heterogeneity, complexity, and reductive environment of TME. To overcome these challenges, various strategies have been developed to enhance the CDT performance, such as combining CDT with other therapies, designing multifunctional nanomaterials, or using bacteria as bioreactors^130^.

CDT can provide excessive levels of ROS that can lead to the exposure of tumor-associated antigens, facilitating the phagocytosis of dead cells and debris by antigen-presenting cells and triggering immune responses throughout the body. This process is known as immunogenic cell death and can enhance the antitumor immunity of CDT. Nanostructures exhibit characteristics akin to those of enzymes, rendering them amenable to integration with bacterial therapeutic approaches for cancer^131^. A recent investigation conducted by Fan et al. shed light upon a bioreactor employing a bacteria-based Fenton-like reaction. Within the confines of this investigation, *E. coli* MG1655 underwent genetic manipulation to induce the overexpression of the respiratory chain enzyme II. This augmentation resulted in an elevated hydrogen peroxide (H_2_O_2_) concentration within the tumor tissues under scrutiny^132^. The process involved the covalent conjugation of magnetic iron oxide nanoparticles to the bacterial surface, resulting in the catalysis of H_2_O_2_ to generate hydroxyl radicals with toxic properties^132,133^. Consequently, this symbiotic combination of bacteria and nanoparticles exhibited remarkable tumor colonization capabilities and autonomously facilitated Fenton-like reactions, thereby impeding the proliferation of tumors in a murine model featuring CT26 tumors.

**Fig. 10:**
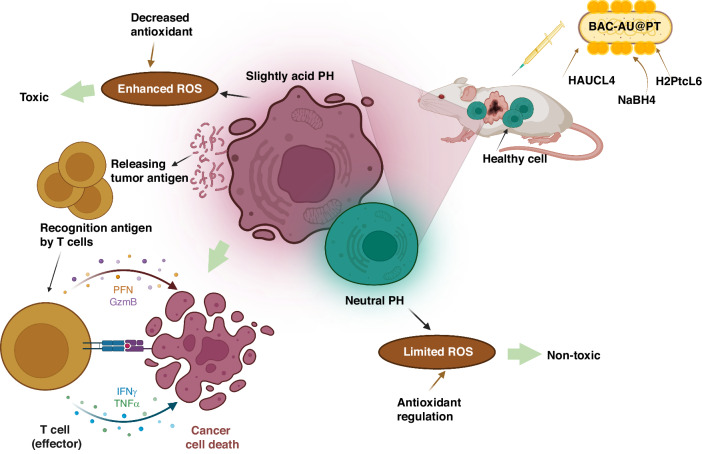
How Bac-Au@Pt nanoparticles can selectively kill cancer cells and activate immune responses. Bac-Au@Pt nanoparticles are made of bacteria and gold coated with platinum. They can produce ROS when they encounter a slightly acidic environment, which is common in tumors. The ROS can damage the cancer cells, which have low levels of antioxidants to protect themselves. The damaged cancer cells can then release signals that attract immune cells to attack the tumor. Normal cells, which have higher levels of antioxidants and a neutral environment, are not affected by the nanoparticles. Adapted from ref. ^134^. ACS Nano, 2021. Created with BioRender.com

A recent research study has divulged the emergence of a groundbreaking multifunctional nano-enzyme system, denoted as Bac-Au@Pt, which is intricately adorned over the exterior of bacterial entities. This system demonstrates a remarkable proficiency delivering reactive oxygen species (ROS) to malignant cells, thereby effectively diminishing the intracellular antioxidant capacity unique to tumors. Furthermore, this system elicits the liberation of interferon-gamma (IFN-γ) from T cells (Fig. 10)^134^. A recent paper delved into the utilization of “nano-factories” as a means to augment the efficacy of cancer therapy. The investigation has successfully devised a pH-responsive nanoscale system denoted as PLNP Cu, which effectively integrates metabolic therapy and immunotherapy with remarkable antitumor effects. The PLNP Cu effectively harnesses the intricate TME to engender the production of pernicious hydroxyl (OH) substances via Fenton-like reactions, instigating the process of immunogenic cell death (ICD) and inciting the immune system to mount a formidable defense against cancer. Furthermore, the utilization of PLNP Cu effectively diminishes the levels of intracellular and extracellular lactic acid, thereby inducing a transformative effect on immunosuppressive TME. This transformative effect is characterized by the polarization of tumor-associated macrophages towards an M1 phenotype, ultimately resulting in an enhanced immune cell response in close proximity to tumor cells^135^. A recent study has revealed that the utilization of an extracellular matrix-degrading STING nano-agonist has shown promising results in stimulating the activation of the STING pathway. This process results in the release of tumor-associated antigens and damage-associated molecular patterns (DAMPs), which can prime the immune system against the tumor. This, in turn, has enhanced the effectiveness of CDT immunotherapy when combined with NIR-II laser treatment. The outcome of this combination has been the eradication of tumor cells and the initiation of immunogenic cell death^136^. Moreover, the STING nano-agonists can promote the uptake and processing of tumor-derived antigens by antigen-presenting cells, such as dendritic cells^136^. The utilization of a nanoscale system facilitates the convergence of controlled drug delivery technology and immunotherapy, thereby engendering a harmonious synergy. This confluence presents a promising approach to achieve remarkable curative efficacy, even when administered at minimal dosages.

### Generation of reactive nitrogen species (RNS)

One notable hallmark of cancer cells resides in the perturbation of the equilibrium among reduction and oxidation reactions, commonly referred to as redox homeostasis. This disruption serves as an enabling factor in the progression and formation of malignant tumors. In contrast to ordinary cellular entities, neoplastic cells often exhibit heightened concentrations of RNS/ROS, including molecular oxygen (O_2_), H_2_O_2_, OH, and nitric oxide (NO)^137^. RNS/ROS demonstrate a dichotomous function in the advancement of cancer: elevated concentrations directly instigate the demise of cancerous cells, while diminished levels can foster the proliferation of tumors and their subsequent dissemination to distant sites^138,139^.

**Fig. 11:**
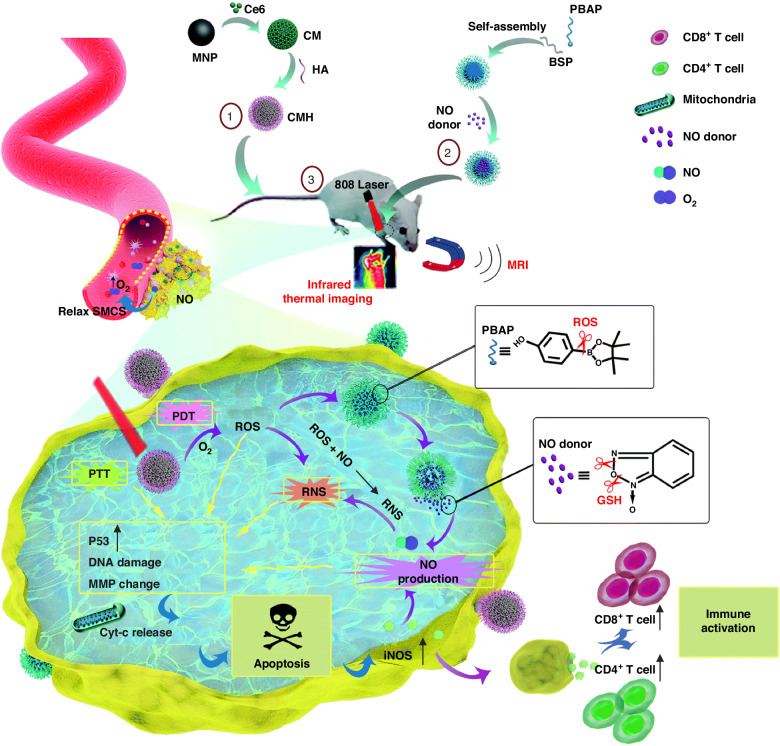
Visual representation of a versatile natural nanoplatform for synergistic therapies, integrating MRI/photothermal imaging guidance and in vivo mechanisms to promote cellular apoptosis and immunotherapy for enhanced antitumor immunity. Adapted with permission from the original source^140^.Biomaterials Science, 2021

A recent research study has successfully showcased the potential of utilizing a fusion of oxidized *Bletilla striata* polysaccharide and chlorine6-melanin-hyaluronic acid nanoparticles microcapsules, which are equipped with NO donors, to facilitate the application of photo-controlled metabolite therapy (Fig. 11). The *Bletilla striata* polysaccharide provides a biocompatible and biodegradable matrix, while the chlorine6-melanin-hyaluronic acid nanoparticles serve as the core of the microcapsules. The incorporation of NO donors within this nanocomposite system allows for the controlled and localized release of NO at the tumor site^140^. The initiation of the release of NO, ROS, and active nitrogen at the sites of tumors is facilitated by the sequential release of ROS and NO. This orchestrated release has the potential to induce apoptosis, a programmed cell death, and stimulate the activation of T cells, specifically CD4^+^ and CD8^+^ cells, thereby impeding the growth of tumors^140^. The ROS generated can directly damage tumor cells, while the NO can inhibit tumor angiogenesis and induce nitrosative stress, leading to cancer cell death. A recent scholarly investigation has elucidated the utilization of carbon nitride (C_3_N_4_) in conjunction with *E. coli* harboring NO generation enzymes for the purpose of photo-controlled bacterial metabolite therapy. When subjected to illumination, the C_3_N_4_ material generates photoelectrons, which can be effectively conveyed to *E. coli*. This transfer facilitates the enzymatic reduction of naturally occurring NO_3_^-^ to the cytotoxic NO compound, resulting in a remarkable 37-fold augmentation. The utilization of C_3_N_4_-infused bacteria exhibited noteworthy suppression of tumor proliferation within a murine model, implying that the activation of immune responses involving dendritic cells and CD8^+^ cytotoxic T cells, may contribute significantly to the anticancer effects alongside the direct generation of cytotoxic NO^120^.

These studies demonstrate the feasibility and efficacy of using photo-controlled bacterial metabolite therapy to manipulate the levels of ROS and RNS in tumor microenvironment. By harnessing the synergistic effects of light, bacteria, and nanomaterials, this novel therapy can achieve selective killing of cancer cells and activation of antitumor immunity. More research is needed to optimize the design and delivery of the photo-responsive systems, as well as to evaluate their long-term safety and biocompatibility. Photo-controlled bacterial metabolite therapy represents a promising strategy to exploit the redox imbalance of cancer cells and to enhance the therapeutic outcomes of cancer treatment.

### Magnetosomes therapy

**Fig. 12:**
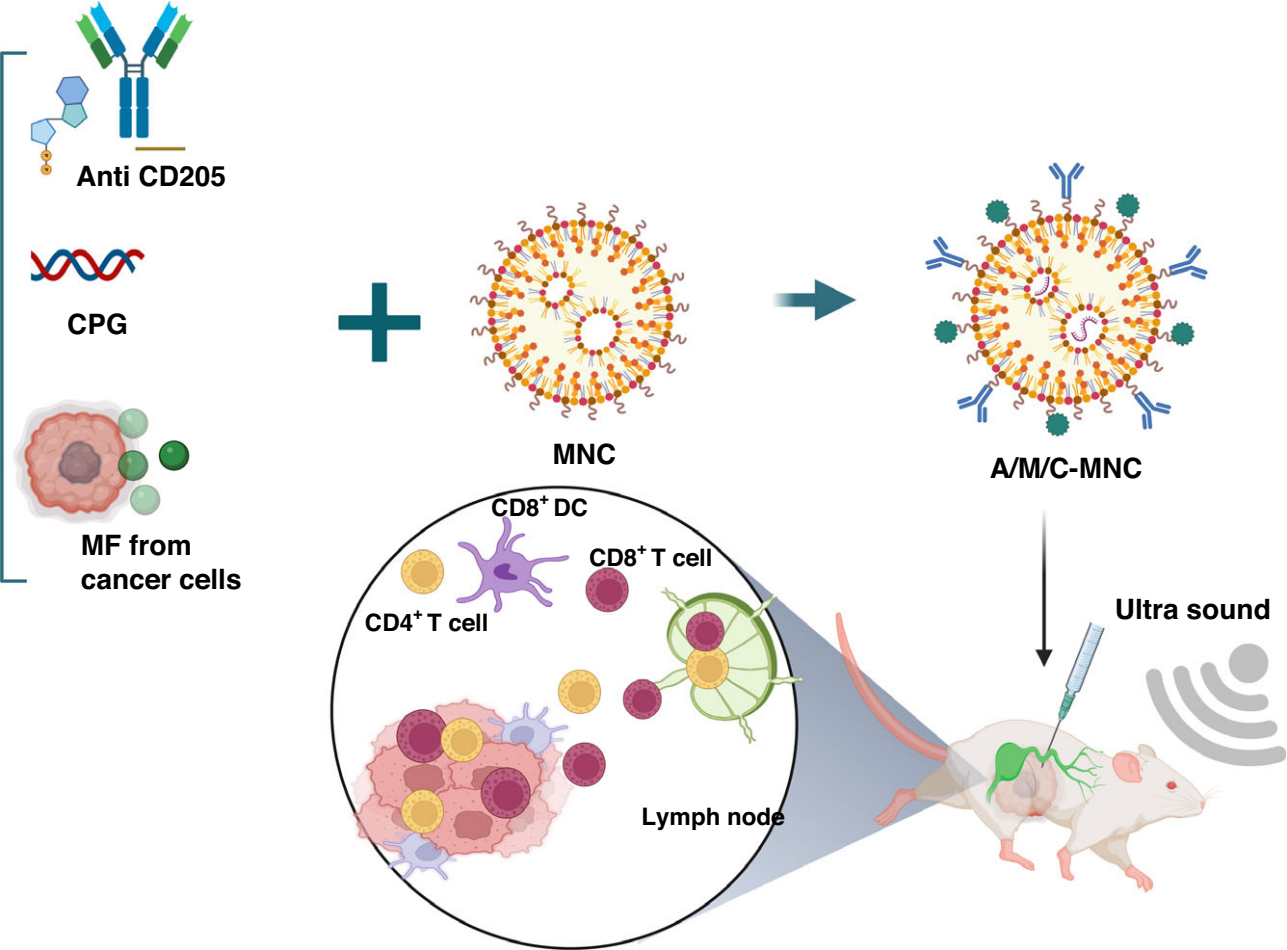
Schematic depiction of fabrication process of A/M/C-MNC. A/M/C-MNCmediated cellular immune responses activate cytotoxicity T lymphocytes (CTLs) and memory T cells (TM cells) for cancer immunotherapy. Created with BioRender.com

In recent years, magnetic bacteria have garnered considerable attention and interest among the scientific community. The magnetic nanoparticles synthesized by these microorganisms, referred to as magnetosomes, have been harnessed by numerous researchers as carriers for pharmaceuticals and genetic material, amenable to manipulation through the application of external magnetic fields. This characteristic renders them a highly appealing candidate for the purpose of remotely manipulating biological hybrid systems^141^. Magnetic nanoparticles, exemplified by superparamagnetic iron oxides (SPIONS), have garnered considerable utilization in diverse realms encompassing both industrial and biomedical domains, owing to their inherent attributes of minimal toxicity, compatibility with biological systems, facile adaptability of surface properties, and responsiveness to magnetic fields^142^. In a prior investigation, the utilization of Fe_3_O_4_ magnetic nano-clusters (MNCs) in conjunction with anti-CD205 was employed to fabricate a cancer vaccine with the remarkable capability of being perceptible within lymph nodes through the utilization of magnetic resonance imaging (MRI). Moreover, the altered anti-CD205 variant facilitates the transportation of immunizations to CD8^+^ dendritic cells and augments the process of cross-presentation of major histocompatibility complex I (MHC I) molecules. This results in heightened proliferation of cytotoxic CD8+ cells (Fig. 12). The findings of the study unveiled that five tumor models exhibited notable efficacy in terms of both preventive and therapeutic interventions^143^. In a separate investigation, a eukaryotic plasmid was formulated with the purpose of encoding heat shock protein 70-Polo-like kinase 1-short hairpin RNA (phSP70-plk1-shRNA). This encoding was carried out under the transcriptional regulation of a heat-sensitive promoter, specifically the human HSP70 promoter, operating at precise ambient temperature conditions. The magnetosomes undergo thermal stimulation through the application of an external alternating magnetic field (AMF), leading to a discernible rise in temperature (reaching 43°C within a span of 3 minutes). This controlled process facilitates the release (DOX) and PHSP70-PLK1-shRNA^144^.

**Fig. 13:**
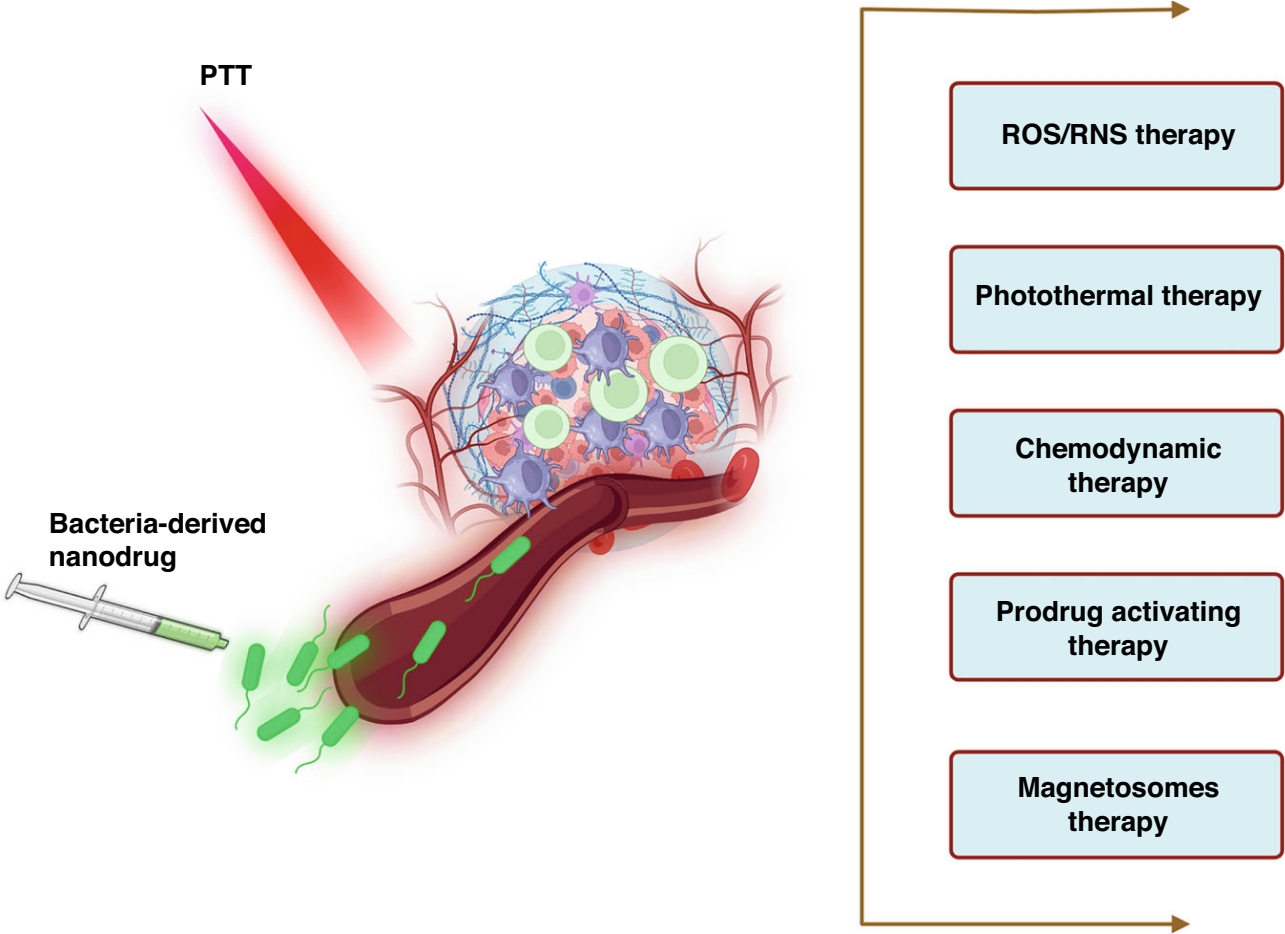
Unveiling the synergistic mechanisms of bacteria-derived nano-drugs in cancer treatment. Created with BioRender.com

On the other hand, a recent investigation was conducted wherein Fe_3_O_4_ magnetic nanoclusters (MNCs) and anti-CD205 were employed to fabricate a cancer vaccine. This innovative approach facilitated the targeted delivery of a greater number of vaccines into CD8^+^ dendritic cells (DC), leading to a heightened proliferation of cytotoxic CD8^+^ cells^145^. The utilization of the anti-CD205 antibody facilitated the specific targeting of the CD205 receptor, which exhibits high expression levels on the surface of CD8+ dendritic cells. Through the conjugation of magnetic nanoparticle carriers (MNCs) with the anti-CD205 antibody, the vaccine formulation demonstrated proficient transport of the immunogenic cargo into the CD8+ dendritic cells. This augmentation led to heightened cross-presentation of tumor-associated antigens on major histocompatibility complex (MHC) class I molecules, thereby eliciting robust activation and proliferation of cytotoxic CD8 + T cells. The outcomes of this investigation elucidated the notable efficacy of the MNC-based cancer vaccine across five distinct tumor models, showcasing both preventive and therapeutic impacts. The precise targeting of the vaccine to the CD8+ dendritic cell subset, pivotal orchestrators of the cytotoxic T cell response, emerged as a critical determinant in the observed antitumor efficacy^145^. A subsequent investigation was undertaken to fabricate a eukaryotic plasmid that encompasses the genetic code for heat shock protein 70-Polo-like kinase 1-short hairpin RNA (phSP70-plk1-shRNA). This genetic construct was placed under the regulatory influence of a heat-responsive promoter, thereby enabling the regulated liberation of DOX and PHSP70-PLK1-shRNA via an externally applied alternating magnetic field (AMF)^144^. The present study harnessed magnetosomes, magnetic nanoparticles synthesized by magnetic bacteria, as a pivotal component of the therapeutic system under investigation. Upon exposure to an alternating magnetic field (AMF), the magnetosomes experienced thermal excitation, resulting in a rapid and controlled elevation in temperature, with levels reaching 43°C within a span of 3 minutes. This orchestrated temperature modulation, facilitated by the magnetosomes, served as a trigger for the activation of a heat-sensitive promoter, thereby enabling the controlled release of the therapeutic cargo comprising doxorubicin (DOX) and a construct targeting Polo-like kinase 1 (PLK1) expression via PHSP70-PLK1-shRNA^144^. Through this integrated approach, the magnetosome-mediated therapy not only engenders suppression of oncogenic PLK1 expression but also orchestrates immune system stimulation, potentially through mechanisms such as magnetic resonance imaging or controlled temperature modulation. This multifaceted therapeutic strategy exhibits significant promise as a cancer treatment modality by effectuating targeted antitumor responses, merging the direct cytotoxic effects of the therapeutic agents with immune system activation^144^. Consequently, it presents considerable value and potential advancement in the landscape of cancer therapy (Fig. 13).

Overall, magnetic bacteria and their magnetosomes have the potential to develop novel and effective strategies for cancer diagnosis and treatment. By combining the advantages of magnetic nanoparticles, such as responsiveness to magnetic fields, biocompatibility, and surface modifiability, with the biological properties of magnetosomes, such as self-assembly, biodegradability, and immunogenicity, magnetic bacteria offer a versatile platform for designing multifunctional nano-carriers that can deliver drugs, genes, or vaccines to specific targets. Furthermore, magnetic bacteria can enable remote control and monitoring of the therapeutic outcomes through the application of external magnetic fields or magnetic resonance imaging. Therefore, magnetic bacteria and their magnetosomes represent a promising avenue for future research and innovation in the field of nano-medicine.

### Hybrid bacterial nano-system for gene therapy

Gene therapy is a promising strategy to treat various diseases, especially cancer, by introducing genetic material into cells to modify their function or expression. However, gene delivery faces many challenges, such as low transfection efficiency, poor stability, and potential immunogenicity of the genetic material. Therefore, there is a need to develop novel gene delivery systems that can overcome these barriers and achieve targeted and controlled gene delivery^146^. One of the emerging approaches is to use bacteria-nanoparticle hybrid as delivery systems. Bacteria can naturally invade cells and release genetic material into the cytoplasm, while nanoparticles can protect the genetic material from degradation and enhance its loading capacity and specificity^17^. Furthermore, it should be noted that the utilization of bacteria-nanoparticle hybrid delivery systems extends beyond the realm of drug administration, as they can also serve as a viable approach for gene therapy^17^. Nevertheless, the achievement of efficient gene delivery necessitates the surmounting of numerous obstacles, including the safeguarding of genetic material against degradation by endogenous nucleases, enhancement of cellular uptake, and successful evasion of the endosomal compartment. Conventional gene delivery platforms, such as mesoporous silica nanoparticles, encounter limitations in effectively transporting nucleic acid molecules into host cells primarily due to their inability to facilitate endosomal escape^147^. Bacteria exhibit remarkable gene delivery capabilities by evading intracellular vesicles through the formation of pores via listeriolysin O^148^. After the genetic material is released into the cytoplasm, it can disperse throughout the nucleus, enabling it to carry out its intended function in the form of plasmid DNA. Likewise, the cytoplasm provides a suitable environment for siRNA to perform its specific role^149^.

**Fig. 14:**
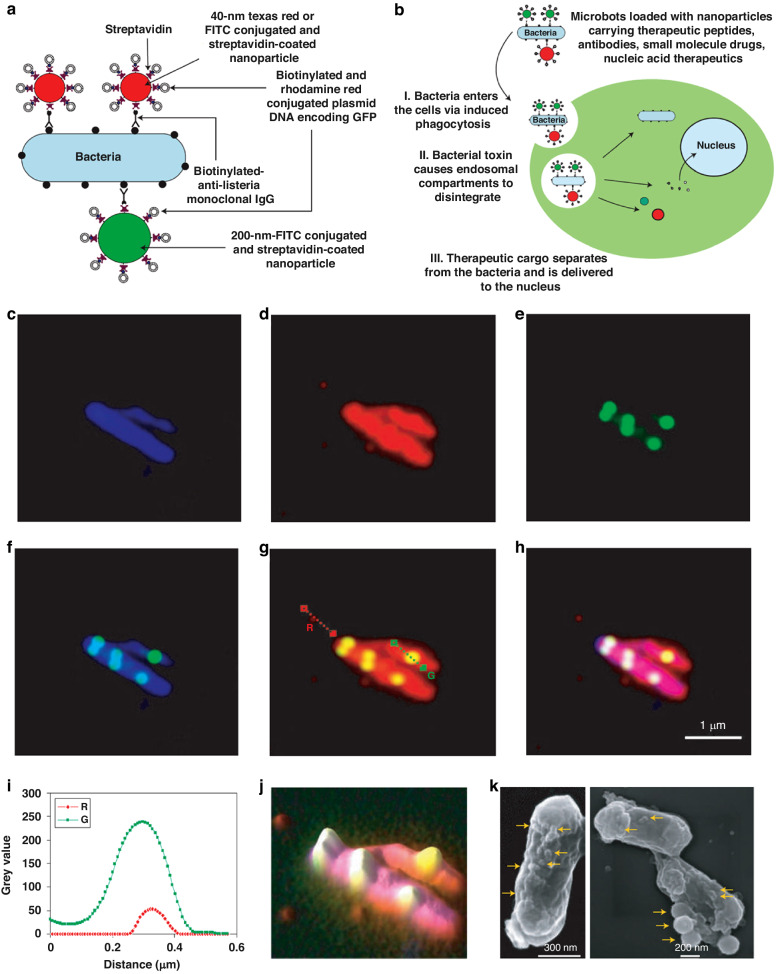
Bacteria-based carriage of nanoparticles and cargo. **a** Interaction between functionalized, multiple-sized nanoparticles and bacteria through biotinylated antibodies and surface-antigen interactions, resulting in the docking of nanoparticles (referred to as microbots). Streptavidin-coated nanoparticles can transport biotinylated cargo. **b** Utilization of microbots for the delivery of intervention agents. **c**–**k** Various examples of assembled microbots with their respective cargos: (**c**) Bacteria (blue), (**d**) Streptavidin-coated 40-nm fluorescent red nanoparticles, (**e**) Neutravidin-coated 200-nm fluorescent-green nanoparticles, (**f**–**h**) Overlay of images: (**f**) Microbots with neutravidin-coated nanoparticles, (**g**) Microbots with streptavidin-coated nanoparticles, (**h**) Microbots with both streptavidin and neutravidin-coated nanoparticles, (**i**) Profiles of lines G (green) and R (red) from image (**g**), (**j**) Simulated height photograph, (**k**) Scanning Electron Microscope (SEM) images of microbots, with arrows indicating the presence of nanoparticles. Adopted with permission from original source^88^

Akin et al. devised a sophisticated bacteria-nanoparticle hybrid delivery system (called microbot) with the purpose of enhancing the efficacy of drug and gene delivery into tumor cells^88^. The researchers employed the conjugation of nanoparticles containing plasmid DNA encoding GFP to bacteria through the utilization of biotinylated antibody and antigen interactions. This intricate process led to the successful preservation of the plasmid within acidic endosomal environments and intracellular enzymes. The hybrid delivery system exhibited remarkable tumor enrichment and a substantial 380-fold amplification of gene expression when compared to a control group that underwent a mock procedure (Fig. 14)^88^. Another example is the use of bacterial ghosts as a vehicle for gene delivery. Bacterial ghosts are empty bacterial cell envelopes that can be loaded with various substances, such as drugs or genes. The utilization of bacterial ghosts as a vehicle for gene delivery holds immense potential in bolstering the immune response against tumors, specifically by targeting dendritic cells (DCs) and facilitating the activation or augmentation of said response through the delivery of tumor-associated antigens (TAAs). This particular methodology has the potential to enhance the transcriptional activity of specific genes that are closely linked to the function of antigen-presenting cells (APCs) as well as malignant tumor cells^51^.

### CRISPR/Cas9: a bacterial nano-system for gene editing of cancer cells

Like many scientific breakthroughs, the CRISPR-Cas9 bacterial nano-system resulted from collaborative research spanning nearly three decades. These studies have elucidated that CRISPR-Cas9 serves as a formidable bacterial immune system, effectively countering viral assaults. Upon the infiltration of bacteria by a viral entity, minute segments of viral DNA are assimilated into the genetic makeup of the bacterial host, causing a cognitive imprint of the assault. This imprint endows the CRISPR-Cas9 apparatus with the capacity to discern and eradicate subsequent viral incursions^150,151^.

In recent years, CRISPR/Cas9 technology has rapidly advanced genome engineering. Since its debut as a genomic editing tool in mammalian cells in 2013^152,153^, CRISPR/Cas9 has continuously expanded its capabilities. It can now not only modify genetic material in cells and organisms^154^, but also induce epigenetic and transcriptional changes^155^. Further, the utilization of CRISPR/Cas9 technology may also be applicable in the context of precise genetic intervention for the purpose of treating tumors. The efficacy of the CRISPR/Cas9 system against tumors is contingent upon the exploitation of a delivery vector, given its distinctive properties. Unfortunately, this requirement presently imposes substantial constraints on the widespread of this technology in cancer treatment. Firstly, it is imperative to acknowledge the inherent instability of the CRISPR/Cas9 system due to enzymatic degradation by serum endonucleases^156^. Furthermore, it is worth noting that the anionic CRISPR/Cas9 exhibits a relatively diminished cellular uptake capacity, primarily due to the presence of electrostatic repulsion forces acting against the negatively charged cell membranes^157^.

In order to achieve optimal tumor accumulation of the CRISPR/Cas9 system, it is imperative that the carrier possesses a circulation time of adequate duration^158^. It is essential that the gene carrier possesses a high degree of efficacy in terms of its ability to penetrate deep into the intricate network of tumor tissues^159^. Furthermore, it is crucial that the chosen vehicle exhibits a sufficiently elevated level of cellular uptake and demonstrates proficient endosomal escape capacity. This is important in facilitating the liberation of the CRISPR/Cas9 system from the confines of the endosome, ultimately leading to the successful delivery of the system into the cytoplasm^160^. Until now, the predominant means of delivering CRISPR/Cas9 systems have chiefly revolved around physical methodologies or viral vectors, thereby imposing constraints on their versatility and posing notable immunological hurdles^161,162^. The successful administration of CRISPR/Cas9 systems into specific tissues continues to be a challenge impeding the widespread implementation of this technique in living organisms^163^. CRISPR/Cas9 sequence exhibits a considerable length that limits gene editing. The intricate spatial conformation of chromosomes can also pose a significant challenge as certain target genes may become less accessible. The fortuitous manifestation of the CRISPR/Cas9 system in non-targeted organs and tissues is also a drawback. Ensuring the safe and effective delivery of the CRISPR/Cas9 system is of utmost importance. In this regard, the use of commercial liposomes for introducing the CRISPR/Cas9 system into cells has shown significant progress and sophistication.

The nano-delivery system, being the most promising vector for the CRISPR/Cas9 system, exhibits a diminished level of cytotoxicity in contrast to commercially available liposomes. Nevertheless, the utilization of the CRISPR/Cas9 system in vivo has proven to be suboptimal. While the intravenous administration of NPs-based CRISPR/Cas9 systems has been explored in limited instances^164,165^, there exists a plethora of untested formulations that are currently under consideration for this promising application. From a discerning perspective, it can be observed that LNPs possess a distinct advantage when it comes to the safety aspect of delivering the CRISPR/Cas9 system in vivo, primarily due to their remarkable biocompatibility. The emergence of tumor microenvironment (TME) responsive liposome nanoparticles (LNPs) has gained significant traction in recent times, owing to the growing recognition of the pivotal role played by TME in the realm of tumor therapy. It should be noted that polymer nanoparticles (PNPs) possess a distinct advantage in terms of their ability to precisely target the delivery of the CRISPR/Cas9 system within living organisms. This can be attributed to their unique physicochemical properties, which render them more amenable to modification by a diverse range of biomolecules^165^. Inorganic nanoparticles (INPs) promise to have a key role in tumor combination therapy. For this reason, their selection as carriers is important when combined with magnetic hyperthermia. The involvement of nanoparticle entities with alternative configurations also assumes significant functions in facilitating the proficient administration of the CRISPR/Cas9 system, as their intricate nano-structure effectively shields it from potential harm. The inherent distinct characteristics exhibited by various types of nanoparticles hold significant merit in the secure and effective transportation of CRISPR/Cas9, both in vivo and in vitro^165^.

Researchers face the challenge of effectively harnessing these inherent characteristics to design appropriate carriers for CRISPR/Cas9. In the meantime, we believe that employing a multifunctional nano-delivery system, which combines various features from different nanoparticles, will be a smarter choice for delivering CRISPR/Cas9 in the future.

In recent years, the combination of nanotechnology with microbial vectors has emerged as a highly promising approach to cancer therapy. This strategy can effectively surmount a multitude of physiological limitations, such as tumor oxygen deprivation, tissue infiltration, and blood-brain barriers. By incorporating light-, self-, and magnetic-driven aspects, this strategy can achieve a significant increase in the anti-tumor effects^166,167^. Bacterial organisms have been studied in cancer therapy for over a century, offering several advantages over synthetic carriers. These benefits include their ability to selectively infiltrate and reside in anaerobic tumors, their genetically manipulable framework for administering therapeutic agents, and their capacity to stimulate the immune system^50^. However, using bacteria for delivery presents challenges, such as assessing risks and ensuring safety. There’s also a need to improve active targeting and expand the range of drugs transportable by bacteria. To address these concerns effectively, considering a nanotechnology-driven approach that leverages bacteria for enhanced tumor therapy is wise.

The strategy of enhancing active targeting and broadening the loaded drugs on bacteria is an example of a typical nanotechnology-driven approach. One potential carrier for this approach is *Lactobacillus*, an anaerobic bacterium that is characterized by its hypoxia metabolic attributes. This distinctive feature endows *Lactobacillus* with the capacity to serve as a carrier for the purpose of selectively targeting solid tumors^49^. Furthermore, the administration of oral live *L. rhamnosus* GG (LGG) in conjunction with immune checkpoint therapy yields a substantial augmentation in the population of dendritic cells (DCs) that elicit the activation of CD8^+^ T cells and their subsequent recruitment into the tumor microenvironment. This strategy proved to inhibit tumor metastasis, growth, and recurrence^168^. Yu et al., have successfully engineered a sophisticated self-propelled multifunctional delivery vector. This vector demonstrates remarkable efficacy in transporting the CRISPR-Cas9 nanosystem, specifically designed for the purpose of downregulating indole amine 2,3-dioxygenase-1 (IDO1). The ultimate goal of this intervention is to enhance the process of immunogenic cell death (ICD) and effectively counteract tumor immune-suppression. *L. rhamnosus* GG (LGG) is a self-propelled and innocuous probiotic that possesses the ability to infiltrate the hypoxic core of tumors, thereby facilitating the effective transportation of the CRISPR/Cas9 system to the tumor vicinity. As a clinical example, Casgevy, a novel cell-based gene therapy, has been approved by the FDA for the management of sickle cell disease in adolescents and adults aged 12 years and above who experience recurrent vaso-occlusive crises. This therapeutic approach represents the first FDA-approved intervention that employs CRISPR/Cas9, a cutting-edge genome editing technology. The treatment involves the modification of patients’ hematopoietic (blood) stem cells using the CRISPR/Cas9 genome editing system^107^.

**Fig. 15:**
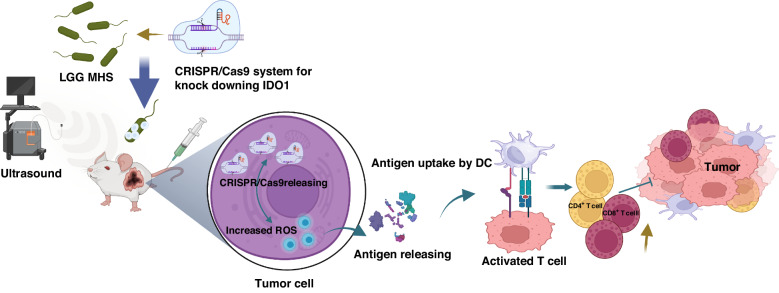
A diagram of how the LGG-MHS nanosystem delivers CRISPR/Cas9 system to reprogram the tumor immune microenvironment (TIME) by stimulating immune response. The nano-system uses ultrasound (US) to trigger the release of Cas9/sgRNA into the tumor cells, where they can edit the genes in the nucleus more effectively. Created with BioRender.com

CRISPR/Cas9 technology enables precise targeting and cleavage of DNA sequences, thereby facilitating accurate editing (removal, addition, or replacement) of DNA at the site of the cut. The modified blood stem cells are then transplanted back into the patient, where they engraft (adhere and proliferate) within the bone marrow. This process leads to an enhanced production of fetal hemoglobin (HbF), a specific type of hemoglobin that enhances oxygen delivery. In individuals with sickle cell disease, increased levels of HbF prevent the characteristic sickling of red blood cells^107^. Whilst LGG exhibits remarkable proficiency in colonizing the neoplastic region, it concurrently elicits a host response that triggers the activation of the immune system^169^. The utilization of the CRISPR/Cas9 nano-system exhibits the capability to generate a substantial quantity of reactive oxygen species (ROS) when subjected to ultrasound irradiation. This phenomenon leads to the induction of immunogenic cell death (ICD). The ROS production may trigger the rupture of endosomal/lysosomal compartments, thereby facilitating the liberation of Cas9/sgRNA^170^, which, enables the effective suppression of the IDO1 gene and the subsequent alleviation of immunosuppressive mechanisms (Fig. 15).

### Integration of bacterial nanotechnology in cancer immunotherapy

#### Checkpoint Inhibitors

Immunotherapy has emerged as a promising method in the treatment of several cancers, with the ability to use the body’s immune system to combat cancer cells. One of the most promising discoveries in this sector is the combination of bacterial nanotechnology with checkpoint inhibitors. This combination treatment attempts to improve immunotherapy efficacy by combining the unique features of bacterial nanoparticles with the immune-modulating effects of checkpoint inhibitors^171^. Despite their different and intricate architectures, these nanoplatforms share four major mechanisms for improving ICB: (1) directing immune checkpoint inhibitors (ICIs) to tumor sites, (2) enhancing tumor immunogenicity, (3) modifying the tumor microenvironment, and (4) pre-sensitizing immune systems^171^. In this sense, nanotechnology, particularly bacterial nanotechnology, has emerged as a promising tool for cancer detection, diagnosis, and therapy, with the potential to overcome immune resistance. Nanomedicines outperform traditional chemotherapeutics in terms of tumoricidal efficacy, adverse effect reduction, and the capacity to increase immune checkpoint blockade (ICB)^172^. Nanotechnology can improve the delivery of immune checkpoint inhibitors (ICIs) to tumors, overcoming obstacles such as poor accumulation at tumor sites and barriers inside cancer tissues. Nanoparticles can efficiently target tumors and even transport ICIs to particular subcellular compartments, increasing their potency^172^. Nanotechnology can improve the efficacy of ICIs by triggering immunogenic cell death and remodeling the immune-suppressive tumor microenvironment, boosting immune cell infiltration, reversing immunological suppression, and activating antigen-specific immune responses against cancer^172^. Nanoparticles can deliver drugs that disrupt immune suppressive pathways, alter the tumor microenvironment, and induce antigen-specific immunity, therefore improving the immune response to cancer^173^.

While bacterial nanotechnologies have been investigated as a delivery vehicle for a variety of anti-cancer medicines^173^, their application as checkpoint inhibitor delivery vehicles has been restricted^172,173^. Gurbatri et al. found that *E. coli* Nissle 1917, which had been modified to make nanobodies against PD-L1 and CTLA-4, was effective in a mouse colorectal cancer model. The synchronized lysis circuit enabled the regulated release of nanobodies during bacterial lysis, resulting in decreased tumor sizes in combination treatment. The toxicity associated with systemic checkpoint blocking was dramatically decreased with bacterial delivery^174^. Another work by Zhao et al. found that *S. typhimurium* harboring PD-1 siRNA accumulated in tumors and increased survival in a melanoma mouse. These research used intratumoral delivery, although oral administration of bacteria is being investigated as a safe and effective delivery route^175^. This method might take advantage of the function of gut bacteria in modifying responses to checkpoint blockade medicines, providing a twofold benefit in cancer treatment.

However, the combination of bacterial nanotechnology with checkpoint inhibitors in immunotherapy appears to be a viable technique for cancer treatment. This combination therapy, which combines the unique features of bacterial nanoparticles with the immune-modulating effects of checkpoint inhibitors, has the potential to improve cancer treatment effectiveness and outcomes. Hence, this method is likely to help produce more effective and customized cancer immunotherapies.

#### CAR-T cell therapy

Cancer treatment has been profoundly transformed by the advent of immunotherapy. Bacterial nanotechnology and chimeric antigen receptor (CAR) T-cell therapy are two immunotherapeutic approaches that have demonstrated encouraging outcomes. CAR-T cell therapy entails the manipulation of a patient’s T cells to produce synthetic receptors that identify and bind to particular tumor antigens, thereby facilitating the precise eradication of malignant cells^176^. Conversely, bacterial nanotechnology enhances anti-tumor immune responses through the delivery of immunomodulatory cargo via engineered bacterial nanoparticles^19^. Nevertheless, the integration of bacterial nanotechnology with CAR-T cells for the purpose of cancer therapy has not been reported yet. As an illustration, nanotechnology is frequently applied to improve CAR-T therapy in this context; for instance, nanoparticles may be utilized as carriers for CAR cargo, or the tumor microenvironment may be modulated^176^. Furthermore, the application of nanotechnology in CAR-T cell immunotherapy typically encompasses novel advancements designed to circumvent obstacles encountered in CAR-T therapy for solid malignancies^176^. In this section, we briefly discuss the limitations of CAR-T therapy in the treatment of cancer, followed by exploring the possibility of combining CAR-T cell therapy with bacterial nanotechnology. Bacterial nanotechnology has emerged as a versatile platform for cancer immunotherapy, as was mentioned in preceding sections. It is possible to manipulate bacteria, including *Salmonella*, *Listeria*, and *E. coli*, in order to encapsulate or produce immunomodulatory molecules on their cell surface^53,148,177^. By targeting and manipulating the microenvironment of the tumor, these bacterial nanoparticles can induce enhanced antitumor immune responses. Despite the notable achievements of chimeric antigen receptor (CAR) T-cell therapy in the treatment of hematological malignancies like diffuse large B-cell lymphoma and acute lymphoblastic leukemia, its implementation in solid tumors has proven to be more difficult due to various limitations:

##### Tumor heterogeneity

Solid tumors frequently exhibit diverse target antigen expression, a characteristic that may result in the formation of antigen-negative tumor cells that evade cytotoxic activity from CAR-T cells^178^. In a clinical trial pertaining to pancreatic cancer, for instance, patients who initially exhibited a favorable response to CAR-T cell therapy subsequently developed resistance as a consequence of the targeted antigen’s loss. This emphasizes the necessity of identifying and targeting multiple antigens within solid tumors in order to thwart escape mechanisms and enhance the efficacy of treatment^179^.

##### Immunosuppressive tumor microenvironment

Factors such as regulatory T cells, myeloid-derived suppressor cells, and checkpoint molecules can substantially inhibit the function and persistence of CAR-T cells in solid tumor microenvironments. For instance, the efficacy of CAR-T cell therapy targeting HER2 in metastatic breast cancer may be compromised due to the loss of HER2 expression in certain tumor cells. Additionally, the efficacy of CAR-T cells may be further impeded by the tumor microenvironment’s elevated concentrations of regulatory T cells and PD-L1^180^.

##### Exhaustion of T cells

Extended exposure to tumor antigens and the immunosuppressive microenvironment of the tumor can result in T cell exhaustion, which is distinguished by diminished effector functionality and heightened expression of inhibitory receptors. For example, HER2-positive breast cancer may impede the efficacy of HER2-targeted CAR-T cells due to the presence of antigen-negative tumor cells and a suppressive microenvironment that is abundant in regulatory T cells and PD-L1^180^.

##### Limited trafficking and infiltration

CAR-T cells frequently encounter obstacles when attempting to infiltrate and traverse solid tumors, which hinder their capacity to identify and eliminate malignant cells. For instance, the dense stromal tissue and absence of blood vessels in pancreatic cancer can impede the penetration of CAR-T cells into the tumor. Additionally, the existence of immunosuppressive cells within the tumor microenvironment, such as myeloid-derived suppressor cells, may facilitate T cell fatigue and further diminish the functionality of CAR-T cells^181^.

##### Financial factors

A considerable number of patients are unable to afford CAR-T cell therapy due to its exorbitant price. The complex manufacturing process and extensive research and development necessary to bring CAR-T cells to market account for the majority of this expense. As a result, a relatively minor proportion of qualified individuals are able to afford this potentially critical medical intervention^182^.

##### Toxicity

In the context of CAR-T cell therapy, neurotoxicity and cytokine release syndrome (CRS) are among the severe adverse effects that may occur. The potential for these effects to be severe and even fatal necessitates the supervision and control of medical professionals. Although there are certain hazards involved, CAR-T cell therapy has demonstrated remarkable efficacy in the treatment of specific malignancies, resulting in substantial patient survival and complete remission. Continuous research and development are essential for expanding patient access to CAR-T cell therapy and enhancing its safety and efficacy^183^.

In this regard, the integration of bacterial nanotechnology with CAR-T cell therapy may potentially offers a promising approach to addressing the limitations of each individual therapy and enhancing the overall efficacy of cancer immunotherapy.

1. Overcoming tumor heterogeneity:
  Bacterial nanoparticles could be engineered to display multiple tumor-associated antigens, allowing CAR-T cells to recognize and target a broader range of tumor cells, reducing the risk of antigen escape.
  The nanoparticles may also be used to deliver a cocktail of tumor-associated antigens, priming the CAR-T cells to recognize and eliminate heterogeneous tumor cell populations^176,179,180,182,183^.
2. Modulating the tumor microenvironment:
  Bacterial nanoparticles could be loaded with immunostimulatory agents, such as cytokines, TLR agonists, or checkpoint inhibitors, and delivered to the tumor site.
  These payloads could help to overcome the immunosuppressive tumor microenvironment by activating and recruiting various immune cells, including dendritic cells, natural killer cells, and T cells, to enhance the anti-tumor immune response^176,179,180,182,183^.
  The nanoparticles could also be designed to selectively target and neutralize specific immunosuppressive factors, such as regulatory T cells or myeloid-derived suppressor cells, to create a more favorable environment for CAR-T cell function 194,196,197,199,200.
3. Preventing T cell exhaustion^176,179,180,182,183^:
  Bacterial nanoparticles could be used to deliver co-stimulatory signals or checkpoint inhibitors to the CAR-T cells, preventing or reversing the process of T cell exhaustion.
  The nanoparticles could also be engineered to deliver metabolic modulators or epigenetic regulators that can enhance the persistence and functionality of CAR-T cells within the tumor microenvironment.
4. Improving trafficking and infiltration^176,179,180,182,183^:
  Bacterial nanoparticles could be designed to express specific homing or adhesion molecules that can facilitate the trafficking and infiltration of CAR-T cells into the solid tumor.
  The nanoparticles could also be used to deliver chemokines or other factors that can attract and guide the CAR-T cells towards the tumor site.

Therefore, by integrating these proposed complementary approaches, researchers would aim to create a synergistic treatment strategy that could overcome the limitations of both CAR-T cell therapy and bacterial nanotechnology, leading to improved clinical outcomes for patients with solid tumors. Hence, by leveraging the unique properties of bacterial nanoparticles, researchers could overcome the limitations of CAR-T cell therapy, such as tumor heterogeneity, immunosuppressive tumor microenvironment, T cell exhaustion, and poor trafficking and infiltration. The combination of these two innovative approaches could lead to synergistic anti-tumor effects, with the bacterial nanoparticles modulating the tumor microenvironment and priming the CAR-T cells for more effective tumor targeting and elimination.

### Bacterial nanotechnology for cancer therapy: from bench to beside

The concept of combining bacteria and nanotechnology for cancer therapy has garnered significant interest in recent years due to its potential to revolutionize the field of targeted drug delivery^70^. Despite the promising preclinical research, there are currently no FDA-approved drugs or clinical trials specifically involving “bacterial nanoparticles” or remedies that integrate bacteria and nanotechnology/nanoparticles for cancer treatment or other diseases. The investigations for effective and targeted cancer therapies has led researchers to explore innovative approaches, including the use of engineered bacteria as carriers for delivering therapeutic agents, such as nanoparticles, directly to tumor sites^70^. This strategy aims to leverage the unique properties of bacteria, such as their ability to preferentially accumulate in hypoxic and necrotic regions of tumors, as well as their capacity for autonomous movement within the tumor microenvironment^35^.

Preclinical studies have demonstrated the potential of using engineered bacteria, such as *Salmonella*, *Escherichia coli*, and *Listeria*, as carriers for delivering various therapeutic payloads, including nanoparticles, to tumor sites. These bacteria can be genetically modified to attenuate their pathogenicity while enhancing their tumor-targeting capabilities^53,148,177^. Additionally, researchers have explored strategies to load nanoparticles onto or within the bacterial carriers, enabling the co-delivery of multiple therapeutic agents^35^. While the concept of “bacterial nanoparticles” or bacteria-nanoparticle remedies holds promise, the search results did not reveal any specific examples of such formulations that have advanced to clinical trials or received FDA approval. The field of bacterial nanotechnology for cancer therapy appears to be still in the early stages of research and development, with ongoing efforts focused on optimizing the bacterial carriers, improving nanoparticle loading and release mechanisms, and addressing potential safety concerns.

The clinical translation of bacterial nanotechnology for cancer therapy faces several challenges that need to be addressed before it can progress to human trials and potential FDA approval. One of the primary concerns is ensuring the safety of engineered bacteria, as their use in human subjects raises questions about potential risks, such as unintended dissemination, immune responses, and potential for reversion to pathogenic forms. Additionally, the development of robust and scalable manufacturing processes for bacterial nanoparticles or bacteria-nanoparticle remedies is crucial for their clinical translation. Maintaining consistent quality, stability, and reproducibility of these formulations is essential for ensuring their efficacy and safety in human trials. Furthermore, the regulatory landscape for bacterial nanotechnology-based therapies presents its own set of challenges. Regulatory agencies, such as the FDA, have established guidelines and requirements for the approval of new drug products, including those involving nanotechnology. However, the integration of bacteria into these formulations may necessitate additional regulatory considerations and rigorous evaluation of potential risks and benefits.

Despite these challenges, the field of bacterial nanotechnology for cancer therapy continues to attract significant research interest due to its potential to address the limitations of conventional cancer therapies. Ongoing efforts are focused on optimizing the design and engineering of bacterial carriers, exploring novel nanoparticle formulations, and conducting comprehensive preclinical studies to evaluate the safety and efficacy of these approaches. As research in this field progresses, it is anticipated that promising candidates for “bacterial nanoparticles” or bacteria-nanoparticle remedies may emerge and advance to clinical trials, paving the way for potential FDA approval and eventual clinical translation. However, this process will require a collaborative effort among researchers, regulatory agencies, and healthcare professionals to ensure the safe and effective implementation of these innovative therapies.

While there are no completed clinical trials or FDA-approved drugs involving “bacterial nanoparticles” or bacteria-nanoparticle remedies, there are ongoing research efforts and early-stage clinical trials that demonstrate the potential of bacterial nanotechnology in cancer therapy. One notable example is a phase 1 clinical trial aimed to evaluate the safety and feasibility of using an attenuated strain of *S. typhimurium* as a carrier for delivery of a therapeutic agent called Azurin to solid tumors. Azurin is a bacterial protein with anticancer properties, and the study investigated its delivery via the engineered *Salmonella* bacteria^184^. The results demonstrated the safety and tolerability of the approach, with no dose-limiting toxicities observed. Additionally, the study provided evidence of bacterial localization within tumors and the potential for therapeutic efficacy, paving the way for further clinical development^184^.

Another ongoing effort is a phase 1 clinical trial that explored the use of an attenuated strain of *L. monocytogenes* as a carrier for delivering a tumor-associated antigen (HPV-16 E7) to induce an immune response against cervical cancer. This trial aims to evaluate the safety, immunogenicity, and preliminary efficacy of the bacterial nanotechnology approach in patients with advanced cervical cancer. While the results are still pending, this study represents a significant step towards translating bacterial nanotechnology into clinical practice^185^.

In addition to these clinical trials, several preclinical studies have demonstrated promising results in using bacteria as carriers for delivering nanoparticles or other therapeutic agents to tumor sites. For instance, one study explored the use of engineered *E. coli* bacteria to deliver gold nanoparticles to tumors in mice. The gold nanoparticles were loaded onto the surface of the bacteria, and the researchers observed successful delivery and accumulation of the nanoparticles within the tumor microenvironment^186^. This approach could potentially be used for photothermal therapy, where the gold nanoparticles are heated by laser irradiation to induce localized tumor cell death^186^. Another preclinical study investigated the use of engineered *S. typhimurium* bacteria as carriers for delivering nanoparticles loaded with chemotherapeutic drugs to breast cancer tumors in mice^187^. The findings showed enhanced tumor targeting and improved therapeutic efficacy compared to conventional chemotherapy alone. This study highlights the potential of bacterial nanotechnology to improve the delivery and efficacy of existing cancer treatments.

However, the translation of these preclinical studies to clinical settings requires rigorous evaluation of safety and efficacy^173^. One of the key challenges in the clinical translation of bacterial nanotechnology is ensuring the safety of engineered bacteria, as their use in human subjects raises concerns about potential risks, such as unintended dissemination, immune responses, and the possibility of reversion to pathogenic forms^173^. To address these safety concerns, researchers have explored various strategies, such as using attenuated or non-pathogenic bacterial strains, incorporating genetic modifications to reduce virulence, and developing robust containment and monitoring systems. For this purpose, in the phase 1 clinical trial the researchers used an attenuated strain of *S. typhimurium* that had been genetically modified to reduce its pathogenicity while retaining its tumor-targeting capabilities^186^.

Another critical aspect of bacterial nanotechnology is the development of robust and scalable manufacturing processes for bacterial nanoparticles or bacteria-nanoparticle remedies^173^. Maintaining consistent quality, stability, and reproducibility of these formulations is essential for ensuring their efficacy and safety in human trials. Researchers are exploring various techniques for loading nanoparticles onto or within bacterial carriers, such as electrostatic interactions, chemical conjugation, or genetic engineering approaches. Furthermore, the regulatory landscape for bacterial nanotechnology-based therapies presents its own set of challenges^18^.

In conclusion, while the search results did not reveal any FDA-approved drugs or clinical trials specifically involving “bacterial nanoparticles” or bacteria-nanoparticle remedies for cancer therapy or other diseases, the field of bacterial nanotechnology holds significant potential for advancing targeted drug delivery and improving treatment outcomes. The ongoing research efforts, early-stage clinical trials, and preclinical studies highlighted in this response demonstrate the progress being made in translating these innovative approaches from bench to bedside. Continued research, addressing safety concerns, and navigating regulatory challenges will be crucial in facilitating the clinical translation of this promising approach.

### Advancements and challenges in the clinical translation of bacterial nanotechnology for cancer treatment

As it was discussed earlier in previous sections, the bacterial nanotechnology, which harnesses the unique properties of bacteria for various applications including cancer therapy, has achieved significant interest. Here are some key aspects related to the clinical translation of bacterial nanotechnology in cancer therapy:

#### Bacteria-based drug delivery

##### Live bacterial carriers

These platforms represent a revolutionary approach to targeted cancer therapy. By genetically engineering specific bacteria strains, scientists can leverage their natural ability to migrate towards tumors. This migration is driven by factors like the unique environment surrounding tumors, which often has low oxygen levels and inflammation^58^. Once these engineered bacteria reach the tumor site, they can be loaded with potent anti-cancer drugs. These drugs can be encapsulated within the bacteria itself or attached to the bacterial surface. The beauty of this system lies in its targeted delivery. Unlike traditional chemotherapy drugs that circulate throughout the body, the bacteria release their cargo directly within the tumor microenvironment. This maximizes the concentration of the drug at the tumor site, enhancing its effectiveness against cancer cells while minimizing potential side effects on healthy tissues^58,144^.

Furthermore, some bacteria can be engineered to not only deliver drugs but also to amplify the anti-tumor response. For instance, the introduction of genes that code for enzymes can be converted inactive prodrugs into their active, tumor-killing forms directly within the tumor. This localized activation strategy can further enhance the potency of the treatment^44^.

However, it’s important to note that this exciting approach is still under development. Researchers are actively investigating different bacterial strains and genetic modifications to optimize tumor targeting, drug delivery, and overall therapeutic efficacy.

##### Bacterial minicells

By spontaneously arising during bacterial cell division, minicells represent a novel and exciting avenue for targeted drug delivery in cancer therapy. These nano-sized (100-500 nm) membrane-enclosed vesicles possess unique properties that address critical challenges in cancer treatment. Firstly, their diminutive size facilitates enhanced penetration into tumor tissues and efficient internalization by cancer cells^188^. This superior cellular uptake compared to traditional delivery systems translates to improved therapeutic efficacy^188^. Furthermore, researchers can leverage the power of genetic engineering to customize the surface of minicells^188^. By expressing specific targeting ligands, such as antibodies, on their exterior, minicells can be directed to bind with high affinity to receptors overexpressed on cancerous cells. This targeted interaction ensures the selective delivery of the therapeutic payload exclusively to malignant cells, minimizing damage to healthy tissues. The therapeutic cargo itself can be remarkably versatile. Minicells can be loaded with potent anti-cancer drugs, RNA interference (RNAi) constructs for silencing oncogenes, or tumor antigens to stimulate a robust anti-tumor immune response^189^.

A significant safety advantage of minicells stems from their inherent lack of a chromosome. Unlike their bacterial counterparts, minicells are rendered avirulent by the absence of chromosomal DNA. This renders them incapable of independent replication, eliminating the risk of uncontrolled bacterial growth within the patient. This characteristic makes them a safer alternative to live, attenuated bacteria used in some cancer treatments. Moreover, the natural origin of minicells as bacterial products minimizes the potential for immunogenicity compared to synthetic delivery systems. Finally, the biocompatible nature of the bacterial cell envelope surrounding minicells allows them to circulate within the body with minimal immune response. This further enhances their therapeutic potential by promoting prolonged circulation and efficient delivery to tumor sites^190^.

Overall, the unique combination of small size, customizable surface engineering, inherent safety profile, and biocompatibility positions bacterial minicells as a highly promising platform for targeted cancer therapy. This innovative approach has the potential to revolutionize cancer treatment by offering a more precise and effective strategy to combat this multifaceted disease.

### Advantages of bacterial nanotechnology

#### Tumor targeting

Bacterial nanotechnology presents a revolutionary approach to targeted drug delivery in cancer therapy by harnessing the inherent tumor-homing properties of specific bacterial strains^50^. This strategy capitalizes on the phenomenon of bacterial chemotaxis, the directed movement of bacteria in response to chemical gradients. The tumor microenvironment, characterized by hypoxia (low oxygen) and inflammation, creates a unique chemotactic signature that attracts motile bacteria strains. This intrinsic targeting mechanism surpasses limitations associated with the Enhanced Permeability and Retention (EPR) effect, which relies on the leaky vasculature of tumors and can lead to off-target drug distribution in healthy tissues^50^. Furthermore, bacterial motility facilitates deeper penetration into tumor regions, overcoming diffusion barriers and promoting a more uniform distribution of therapeutic payloads. Notably, bacterial nanotechnology can be further enhanced by engineering bacteria to act as carriers for drug-loaded nanoparticles. This synergistic approach enables the targeted delivery of a diverse arsenal of therapeutic agents, including potent cytotoxic drugs, gene silencing constructs for oncogene knockdown, and immunostimulatory molecules to activate anti-tumor immune responses^19^. By leveraging these unique capabilities, bacterial nanotechnology holds immense potential for the clinical translation of personalized cancer therapies with improved efficacy, reduced systemic toxicity, and enhanced patient outcomes^44^.

#### Biocompatibility and biodegradability

In the clinical translation of bacterial nanotechnology for cancer therapy, a paramount consideration lies in the biocompatibility and biodegradability of select bacterial strains. These properties are fundamental attributes that contribute to the safety and efficacy of this innovative approach. Certain bacterial strains exhibit inherent biocompatibility, signifying their ability to interact with biological systems without eliciting unwanted immune responses or exerting toxic effects. This characteristic is crucial for cancer therapy, as it ensures the body tolerates the presence of bacteria utilized as drug delivery vehicles. Biocompatible bacteria can navigate biological barriers within the body and reach targeted tumor sites without causing harm to healthy tissues^44,55^.

Moreover, equally significant is the biodegradability of these bacterial strains. After successfully delivering the therapeutic payload to the tumor site, these bacteria can undergo natural degradation and elimination by the body’s metabolic processes. This feature is essential for minimizing any potential long-term effects or unwanted accumulation of bacteria post-treatment. The ability of these bacteria to biodegrade within the body ensures they do not persist beyond their intended function, further enhancing the overall safety profile of bacterial nanotechnology in cancer therapy^58^.

Overall, the synergistic combination of biocompatibility and biodegradability in specific bacterial strains offers a dual advantage in targeted drug delivery for cancer treatment. Their capacity to navigate towards tumors, deliver therapeutic agents, and then be safely eliminated underscores the immense potential of bacterial nanotechnology as a promising strategy in the fight against cancer.

### Challenges and considerations for clinical translation

#### Safety

Strategies to prevent uncontrolled bacterial growth within the body are essential to avoid unintended consequences. Attenuation techniques, such as modifying specific bacterial functions to weaken their virulence, play a critical role in rendering bacteria avirulent and limiting their replication^44,55^. Additionally, the incorporation of “suicide genes” provides a fail-safe mechanism that can be remotely activated to eliminate the bacteria post-therapeutic delivery, ensuring precise control over their presence. In this context, minimizing the risk of bacterial dissemination beyond the intended tumor site is a key consideration in ensuring safety^58^. Selecting bacteria with limited motility or utilizing auxotrophic strains that rely on specific nutrients only available in the tumor microenvironment for survival helps confine their activity. Moreover, employing physical containment strategies like encapsulation in biocompatible hydrogels offers an additional layer of control, restricting bacterial movement within the body and enhancing localized therapeutic effects^173^. Moreover, managing the potential for mutations and horizontal gene transfer is crucial to prevent the development of antibiotic resistance or increased virulence^53^. Utilizing well-characterized, genetically stable bacterial strains and implementing stringent containment measures are vital steps in minimizing these risks. By ensuring the genetic integrity of the bacteria and controlling their interactions within the body, researchers can mitigate the potential hazards associated with genetic modifications and horizontal gene transfer, safeguarding the efficacy and safety of bacterial nanotechnology in cancer therapy^99^. By integrating these detailed safety measures into the clinical application of genetically modified bacteria for cancer treatment, researchers can establish a robust framework for the controlled and secure utilization of bacterial nanotechnology, advancing the development of targeted and efficient therapeutic strategies in the fight against cancer.

#### Immunogenicity

The immune system’s recognition of bacteria, while beneficial in combating infections, poses a significant challenge in the context of bacterial nanotechnology for cancer therapy. This is because the immune system may perceive therapeutic bacteria as foreign invaders, leading to an immune response that could reduce the effectiveness of the therapy or cause inflammation^54^.

To circumvent this issue, researchers are investigating the use of non-immunogenic bacterial strains. These are strains that have been genetically engineered to evade detection by the immune system, thereby enhancing their survival and therapeutic effect. However, this approach must be carefully balanced. While non-immunogenic bacteria can evade the immune system, they may not stimulate a sufficient anti-cancer immune response^57^.

Another strategy being explored is the cloaking of bacteria with biocompatible materials. This approach, often referred to as the ‘stealth’ approach, can prevent the immune system from recognizing the bacteria, much like how some pathogens evade immune detection. This strategy can enhance the survival and effectiveness of the therapeutic bacteria, but it also needs to be finely balanced to ensure that it does not completely inhibit the beneficial anti-cancer immune response^2^.

Moreover, the advent of nanotechnology has led to the development of nanoparticles that can be used to encapsulate and deliver therapeutic agents to cancer cells. These nanoparticles can be designed to have selective binding capacity, high permeability, and retention effect, which can enhance their effectiveness in cancer therapy. Furthermore, the interaction between these nanoparticles and immune cells can be exploited for a sustained anti-tumor effect^17^.

However, the clinical translation of these strategies faces several challenges. These include ensuring the biocompatibility and non-toxicity of the nanoparticles, achieving efficient cellular internalization, and accurate subcellular localization. Therefore, future research in this field will likely focus on overcoming these challenges to maximize the therapeutic outcomes of bacterial nanotechnology in cancer therapy^132^.

#### Regulatory hurdles

Regulatory hurdles in the clinical translation of bacterial nanotechnology for cancer therapy are multifaceted and complex. One of the primary challenges relates to the biocompatibility and bioaccumulation of nanoparticles. The body’s immune response to foreign substances can lead to the rapid uptake of nanoparticles by the liver, reducing their availability for therapeutic purposes. Moreover, nanoparticles can have low chemical stability in blood circulation, leading to their premature degradation and loss of therapeutic effect^8^.

Another significant hurdle is the non-specific absorption of nanocarriers. Nanocarriers are designed to deliver therapeutic agents directly to cancer cells, but they can also be absorbed by healthy cells, reducing their therapeutic efficacy and potentially causing unwanted side effects. Overcoming this challenge requires a combination of rational nanocarrier design and a fundamental understanding of tumor biology^11^.

Furthermore, the distinct pharmacodynamic (PD) and pharmacokinetic (PK) profiles of nanomedicines compared to their associated constituent materials and payloads pose additional regulatory challenges. These differences can affect the safety, efficacy, and dosage requirements of nanomedicines, necessitating extensive preclinical and clinical testing^2,11^.

Moreover, the clinical testing and development of cancer medicines face several challenges. These include ensuring the biocompatibility and non-toxicity of the nanoparticles, achieving efficient cellular internalization, and accurate subcellular localization. Overcoming these challenges requires innovative approaches and rigorous testing methodologies^13,35^.

In addition to above, the clinical development and regulatory approval of bacterial medications will encounter significant obstacles due to the absence of existing products and the potential negative perception of using bacteria as medicinal agents. To address societal issues, it is imperative to gain patient acceptability by implementing outreach efforts and advancing technology. While it is probable that their medical use would be approved for treating life-threatening ailments, it is crucial to exercise caution in order to avoid establishing detrimental precedents that could hinder the advancement of the profession. Furthermore, the successful implementation of this technology into the market will require overcoming production obstacles. The integration of synthetic biology and nanomedicine will enhance the creation of more effective delivery methods, ensuring both safety and efficacy. This will further accelerate the clinical implementation of medicines based on bacteria^191,192^.

### Overcoming key challenges for clinical success

The challenges of clinical implementation of bacterial nanotechnology are still the same issues that arise when using bacteria in clinical settings. Despite ongoing clinical translational activities, drug development in the bacterial nanotechnology field which uses live bacterial species presents particularly difficult challenges both for investigators and regulatory authorities^191,192^.

First, the use of live bacteria in bacterial nanotechnology cannot be regulated in the same way as conventional drugs, while heating or filtering cannot be used to sterilize live bacteria. If the bacteria are to be used as therapeutic agents, it is crucial to maintain axenic cultures and eliminate potentially harmful substances, such as bacterial pathogens. Moreover, the viability of therapeutic bacteria should be guaranteed (without batch-to-batch variation), regardless of any upscaling (to facilitate production on an industrial scale), packaging, shipping, or storage methods^55,188^.

Second, determining the appropriate dose and schedule for nano-bacterial setting is also challenging because bacteria may not follow conventional pharmacokinetics and may have a different dose-response relationship than other drugs. Effective doses may be related more closely to the quality of the target tumor rather than to the bacterial dose administered^19^.

Third, one of the major concerns in the field of bacterial nanotechnology is the toxicity of bacteria due to associated toxins, which may lead to serious infections, considerable side effects, and even death^19^. Researchers are, therefore, using attenuated and genetically modified strains to overcome these adverse outcomes. Reducing or removing specific virulence factors from bacteria by genetic modifications can also remedy the toxicity associated with using bacterial nanotechnology. However, it should be noted that there is a tradeoff between reducing virulence and removal of virulence factors and clinical outcomes, as removing the virulence of a bacteria can reduce the potency of its anti-cancer effects^53,193,194^. It is well documented that bacterial strains manipulated for cancer therapies are sensitive to changes in their virulence factors. Microbe-associated molecular patterns (MAMPs) need additional attention when they are adapting to bacterial strains during cancer therapy. However, it has been previously reported that structural changes in LPS can cause changes in the physiology of bacteria to transform from a virulent strain to a strain with anticancer properties. For example, a change in the structure of lipid A to hexa-acylated lipid A has led to increased affinity for Toll-like receptor 4 (TLR4), which can induce anti-cancer responses. Another major challenge in this field is the short half-life of the bacterial peptide of protein and unstable DNA^191,192^.

Additionally, it must be noted that it is not suitable for patients who have been on certain types of chemotherapy, as these may suppress the immune system to the extent that it cannot sufficiently respond to bacterial colonization. Additionally, live bacterial products can colonize foreign bodies like artificial heart valves, joint replacements, and implanted medical devices, which may serve as reservoirs for infection. Furthermore, recombinant plasmids carried by bacteria can be mutated, thus changing the fate of anti-tumor action before the cancer cells are penetrated. This can lead to various associated risks, including therapy failure, infection, or death. A major public health concern is the development of multi-drug resistance of many of the bacteria used in bacterial nanotechnology^185,193,194^.

Fourth, tumor-targeting bacteria have peculiar distinctive features including unique gene packaging, targeting the hypoxic environment of tumors, and tumor selectivity, which make them an ideal vehicle for delivering therapeutic cargo specifically targeting cancers of various origins^195^. However, although engineered bacteria have gained a high therapeutic potential to target tumors, due to the high heterogeneity of cancers at the molecular and histologic levels, a single anti-cancer agent may not be able to achieve a cure by itself. The combinatorial approach which combines nanomaterials with bacterial species may be required to develop a promising anti-cancer therapy^191,192^.

The minimum available FDA guidance documents related to bacterial nanotechnology are “Microbial Vectors Used for Gene Therapy” (September 2016) and “Preclinical Assessment of Investigational Cellular and Gene Therapy Products” (November 2013). Moreover, it is recommended that potential sponsors of investigational new drugs contact the FDA to obtain additional guidelines before submission^191,192,195^.

The challenges of clinical implementation of bacterial nanotechnology are still the same issues that arise when using live bacteria in clinical settings^188^. Despite ongoing endeavors in clinical translation, drug development within the realm of bacterial nanotechnology, employing live bacterial species, presents notably formidable challenges for both investigators and regulatory bodies. Primarily, the utilization of live bacteria in bacterial nanotechnology eludes regulation akin to conventional drugs, as standard methods such as heating or filtration cannot ensure the sterilization of live bacteria. For these bacteria to serve as therapeutic agents, it is imperative to maintain axenic cultures and eliminate potentially hazardous substances, such as bacterial pathogens^189^. Furthermore, ensuring the viability of therapeutic bacteria, devoid of batch-to-batch variation, is essential, irrespective of upscaling efforts aimed at facilitating industrial-scale production, packaging, shipping, or storage methods^191,192^. Secondly, determining the appropriate dosage and regimen for the nano-bacterial setting poses a challenge, given that bacteria may not conform to conventional pharmacokinetics and may exhibit a distinct dose-response relationship compared to other pharmaceuticals^191,192^. Effective doses may be more closely correlated with the characteristics of the target tumor rather than the administered bacterial dose^11^. Thirdly, a significant concern in the field of bacterial nanotechnology pertains to bacterial toxicity stemming from associated toxins, potentially resulting in severe infections, notable side effects, or even fatality^11^. To mitigate these adverse outcomes, researchers are exploring attenuated and genetically modified strains. However, there exists a tradeoff between reducing virulence and clinical outcomes, as diminishing virulence may compromise the anti-cancer efficacy of bacteria. Notably, bacterial strains engineered for cancer therapy are susceptible to alterations in their virulence factors, warranting careful consideration of microbe-associated molecular patterns during cancer therapy^175,185,194^. Moreover, the short half-life of bacterial peptides or proteins, along with unstable DNA, presents an additional challenge in this domain. Furthermore, patients undergoing certain types of chemotherapy may not be suitable candidates for bacterial nanotechnology due to potential immunosuppression, which may impede their ability to mount an effective response to bacterial colonization^175,185,194^. Additionally, live bacterial products have the propensity to colonize foreign bodies, such as artificial heart valves or implanted medical devices, potentially serving as reservoirs for infection. Fourthly, tumor-targeting bacteria possess distinctive features, including unique gene packaging and the ability to target the hypoxic environment of tumors, rendering them an ideal conduit for delivering therapeutic cargo aimed specifically at various cancer types. However, the high heterogeneity of cancers at molecular and histologic levels necessitates a combinatorial approach, integrating nanomaterials with bacterial species, to develop a more efficacious anti-cancer therapy^175,185,194,196^.

## Conclusion and future perspective

Bacterial nanotechnology rapidly advanced due to its unique advantages: bacteria’s ability to selectively target and reside in tumors, abundant innate antigens, and impressive engineering capabilities. However, the complexity of bacteria and their living nature poses risks in tumor treatment. Bacteria and their components possess strong immune-stimulatory qualities, but challenges like limited component extraction, dosing, and delivery may hinder their widespread use in comprehensive anti-tumor immune responses. Additionally, the variability of bacterial immunotherapy should be noted. To prevent severe adverse reactions, the careful management of microorganisms and their components is crucial to avoid inflammation and toxicity^11^.

Bacteria can encapsulate genetic material, allowing them to produce a variety of therapeutic proteins and alter their gene networks to fight cancer. However, several issues should be addressed before clinical translation begins. These include determining the appropriate dosages of pharmaceuticals, microorganisms, submicroscopic particles, and hereditary data, as well as assessing nano-scale substances’ resilience in various physiological settings and refining the method of combining them^58^.

Clinical trials should also determine bacterial nano-therapeutics’ efficacy, safety, and replicability. These nano-drugs can stimulate different immune cell responses in the living organism, resulting in favorable anticancer results; however, their TME dispersion may limit their efficiency. Thus, combining bacterial nano-drugs with immunotherapy, radiation, PTT, PDT, CDT, ROS/RNS therapy, prodrug activating therapy, and magnetosomes therapy are possible improvements in treatments^13^.

Nanostructures, with their appealing physical and chemical properties, hold significant promise for cancer treatment using bacterial therapies. However, this field presents challenges and opportunities. Enhancing therapies like photocatalytic, photothermal, and nanomaterial-based catalytic treatments can boost effectiveness. The enzymatic properties of nanomaterials may be less stable in various physiological conditions, affecting their therapeutic potential. Nevertheless, methods like PTT, PDT, CDT, and ROS/RNS therapies show substantial biomedical potential despite these hurdles^191,192^.

Rapid advancements in synthetic biology and chemistry have led to over a century of research into bacterial-mediated drug delivery systems (DDS). Bacteria can efficiently pass through physiological barriers and infiltrate tumor tissues, exhibiting superior homing capabilities compared to nanoparticles relying on enhanced permeability and retention (EPR). Various modification strategies have enhanced the safety and therapeutic effectiveness of these systems, positioning them as promising candidates for cancer treatments and drug delivery. According to this systematic review, bacterial nanotechnology is poised to play an increasingly significant role in cancer treatments, expanding the spectrum of anticancer medications. Building on prior research, genetic manipulation can enable bacteria to produce chemicals within the hypoxic tumor microenvironment, potentially enhancing the effectiveness of nano-material-encapsulated chemotherapeutics^197^.

Despite preclinical advances in DDS caused by bacteria, several barriers prevent its clinical application. Bacteria-centric DDS poses risks that worry users. This requires culturing bacteria with improved safety and nanoparticle conjugation. These systems’ safety measures must also be assessed. Controllability, repeatability, and stability of preparation methods must be investigated. These methods consider binding site, conjugated bacteria and nanoparticles, and conjugation intensity^191,192^.

The bacterial drug delivery system (DDS) lacks comprehensive data on various crucial aspects, including stability, metabolism, clearance, drug loading, in vivo retention, pharmaceutical stability during storage, pharmacokinetics, biodistribution, and adherence to current good manufacturing practices (cGMP)^44,55,56,125^. Addressing this knowledge gap requires further research. Given the inherent complexities and potential risks in this field, clear standards and manufacturing protocols for this dynamic medication system are still missing. To establish such standards, collaboration between bioengineering and complex biological processes is essential. Further research into intracellular mechanisms and cellular distribution will aid in the development of regulatory guidelines^44,55,56,125^. Additionally, funding for improved in vitro characterization methods is necessary to investigate the DDS’s pharmacokinetics in both in vitro and in vivo settings, ultimately advancing its clinical application^44,55,56,125^.

Nanotechnology-facilitated medication and gene delivery systems using bacterial organisms provide novel therapeutic possibilities. Nanomaterials help these hybrid bacterial nano-systems deliver drugs and genetic material to tumors. These hybrid systems show synergistic cancer treatment efficacy while having no effect on the bacterium’s targeting ability. However, careful bacterial strain curation improves drug-targeting mechanisms^44,55,56,125^. Felfoul et al. conjugated drug-loaded liposomes with *Magnetococcus marinus* strain MC-1^25^. Using magnetic forces, this clever method infiltrated hypoxic areas of HCT116 colorectal xenografts. Hybrid bacterial nano-systems can boost the therapeutic index of many small-molecule medicines in tumor hypoxic areas^25^. Thus, to fully realize their cancer-treating efficacy, bacteria-nanoparticle hybrid systems must be rationally formulated.

While bacterial nanotechnology holds promise for the future, several critical issues must be addressed before its clinical use. Managing bacterial membrane components is essential to prevent systemic inflammation, latent inflammation, and toxicity. Rigorous testing is required to assess therapeutic effectiveness and reproducibility. Hybrid nanosystems need to consider various factors, including bacterial and nanoparticle quantities, pharmaceutical agents, genetic data, and the method of combining nanoparticles with bacteria. Nanotechnology has ushered in a new era in cancer therapy centered on bacteria, offering innovative approaches to enhance clinical cancer treatment. Nano-bacterial therapy, particularly with improved tumor-targeting bacteria, is expected to emerge as a potent tool in the battle against cancer.
